# Ictal semiology in lateral temporal epilepsy: A systematic review and meta‐analysis

**DOI:** 10.1002/epd2.70189

**Published:** 2026-02-02

**Authors:** Jakob I. Doerrfuss, Georg Zimmermann, Martin Holtkamp

**Affiliations:** ^1^ Department of Neurology with Experimental Neurology Charité – Universitätsmedizin Berlin, Corporate Member of Freie Universität Berlin and Humboldt‐Universität Zu Berlin Berlin Germany; ^2^ Department of Neurology, Neurological Intensive Care and Neurorehabilitation, Christian Doppler University Hospital, Center for Cognitive Neuroscience, Member of the European Reference Network EpiCARE Paracelsus Medical University Salzburg Austria; ^3^ Epilepsy‐Center Berlin‐Brandenburg Institute for Diagnostics of Epilepsy Berlin Germany

**Keywords:** anatomo‐clinical correlation, epilepsy surgery, focal epilepsy, ictal semiology, lateral temporal epilepsy

## Abstract

**Objective:**

We performed a systematic review of the ictal semiology of the *lateral temporal lobe* in focal epilepsy aiming to summarize the state‐of‐the‐art anatomo‐clinical correlations in the field and help guide interpretation of ictal semiology within the framework of pre‐surgical evaluation.

**Methods:**

After preregistration of the study protocol (PROSPERO‐ID CRD42024498889), we used the PRISMA‐based approach and systematically searched PubMed and EMBASE for relevant literature on the semiology of lateral temporal lobe epilepsy (TLE). A random‐effects meta‐analysis with the inverse variance method was used to calculate pooled estimates.

**Results:**

Six studies with 94 patients fulfilled our inclusion criteria. All studies also included patients with TLE confined to other neocortical temporal structures without performing analyses specifically on lateral temporal structures. The most common signs comprised oral automatisms, manual automatisms, and behavioral arrests; however, these likely do not manifest at seizure onset but during propagation. Overall, GRADE evaluation indicated very low evidence for any signs or symptoms being associated with anatomic localization. We performed a meta‐analysis of the diagnostic accuracy of the presence or absence of relevant ictal signs and symptoms for differentiating seizures in lateral versus mesial TLE. The presence of auditory auras was highly specific for lateral TLE (.98 [95% CI .88–1.00]) with an overall sensitivity of .06 [95% CI .02–.22]. Lack of epigastric or olfactory/gustatory aura was associated with a high sensitivity for differentiating lateral from mesial TLE (.93 [95% CI .80–.98] and .96 [95% CI .84–.99], respectively); specificity was .38 [95% CI .26–.51] for lack of epigastric aura and .13 [95% CI .06–.25] for lack of olfactory/gustatory aura.

**Significance:**

The six studies included in this systematic review defined the boundaries of the lateral temporal lobe quite heterogeneously and only about two‐thirds of patients unambiguously had lateral temporal lobe epilepsy likely explaining the wide spectrum of ictal semiologies. Furthermore, there is often no differentiation into seizure onset versus propagation. Auditory auras represent the only specific semiology for lateral TLE, though this symptom occurs in less than every 10th patient. Comparing lateral versus mesial temporal lobe epilepsy, the presence of epigastric auras and even more so of olfactory/gustatory auras indicates mesial rather than lateral seizure onset.

**Key points**

- The anatomical delineation of the lateral temporal lobe is imprecise.
- Auditory auras are highly specific for seizures originating from the lateral temporal lobe; however, they occur in fewer than 10% of patients with lateral temporal lobe epilepsy.
- The occurrence of olfactory and gustatory as well as epigastric auras argues against lateral temporal seizure onset and instead hints to the mesial temporal lobe.

## 1 INTRODUCTION

Penfield and Jasper have reported in the 1950s that intraoperative electrical stimulation of the lateral temporal lobe, and more specifically of the transverse gyrus of Heschl, produces an auditory sensation.^1^ These responses have been evoked especially when an auditory aura was part of the habitual epileptic seizures. In the 1989 proposal for a Classification of Epilepsies and Epileptic Syndromes of the International League Against Epilepsy (ILAE), temporal lobe seizures were dichotomized into those originating in amygdalo‐hippocampal structures and those with onset in the lateral temporal lobe.^2^ Ictal signs and symptoms from the latter region comprised auditory hallucinations, dreamy states, visual misperceptions, language disorders, and the notion that seizures may progress to what was termed at that time “complex partial” seizures, that is, following current ILAE terminology, focal impaired consciousness seizures.^3^

This simple dichotomization reflects the problem with the use of unambiguous anatomical boundaries of the lateral temporal lobe. Some authors define the lateral temporal lobe as all neocortical parts of the temporal lobe, that is, all extra‐amygdalo‐hippocampal temporal structures.^4^, ^5^, ^6^, ^7^ Other authors use a more strict definition, separating the lateral temporal lobe from adjacent temporo‐basal and ‐polar structures.^8^ In this systematic review, we aim to use these strict anatomical boundaries of the lateral temporal lobe.

Within the framework of the Paros Epilepsy Meeting in April 2024, we thus performed a systematic review of the ictal semiology of the lateral temporal lobe with the view to summarize the state‐of‐the‐art anatomo‐clinical correlations in the field and help guide interpretation of ictal semiology in pre‐surgical evaluation.

## 2 METHODS

### 2.1 Search method and eligibility criteria

We conducted the systematic review of the published evidence and reported its results according to the Preferred Reporting Items for Systematic Review and Meta‐Analysis (PRISMA) statement.

The protocol for this systematic review was registered in the PROPSPERO database for systematic reviews before the literature search began (ID: CRD42024498889).

We searched in PubMed and EMBASE using the following string: ((lateral OR neocortical) AND (temporal)) AND (epilep* OR seizure*) AND (surg* OR EEG) AND (video). The date last searched was January 3, 2024. The EMBASE search was conducted via OVID.

We selected studies published as papers in peer‐reviewed journals, without language limitations. Studies that consisted only of an abstract or a poster were not included. Two independent reviewers (JD and MH) screened titles, abstracts, and full‐text articles for eligibility criteria. Disagreements at the full‐text screening phase and the data extraction phase were solved by consensus.

We selected series including at least three patients.

### 2.2 Data extraction

For each selected publication, we extracted the number of reported patients, the proportion of patients with informative data regarding the topic of this review, and all such data informing on anatomo‐clinical correlations. We evaluated the risk of bias of each publication using a QUADAS2‐adapted assessment:

**Risk of selection bias:**

- Was a consecutive or random sample of patients enrolled?
- Was a case–control design used?
- Did the study avoid inappropriate exclusions?

- Could the selection of patients have introduced bias?

**Risk of assessment bias:**

- Was semiology interpreted blinded to other data?

**Reliability of the reference standard:**

We further assessed our level of confidence in the reported epileptogenic zone (EZ) according to a recently developed method. The latter is based on the availability and findings from MRI, intracerebral EEG and post‐operative outcome, and distinguishes four levels of evidence (very high, high, moderate and low) defined as follows:

1. “very high” confidence in the reported EZ for patients with Engel class IA after at least 1 year of post‐operative follow‐up
2. “high” confidence in the reported EZ for patients with either: (i) a well‐delineated focal lesion suspected to represent at least part of the EZ (according to the authors of the publication), (ii) a well‐delineated EZ according to all available iEEG data (according to the authors of the publication), or (iii) an Engel class I (but not specified IA) after at least 1 year of post‐operative follow‐up;
3. “moderate” confidence in the reported EZ for patients with MRI signs of hippocampal sclerosis or atrophy suspected to be at least part of the EZ;
4. “low” confidence in the reported EZ for patients whose MRI would be normal or show multilobar, multifocal, or poorly delineated lesion, or with a poorly delineated EZ according to all available iEEG data (according to the authors of the publication), or an Engel class II–IV post‐operative outcome provided the preoperatively presumed EZ has been entirely removed. Surgical failure in patients whose suspected EZ would not have been fully removed would not be considered for grading.

If several of the above items provided different levels of confidence, the post‐operative outcome prevailed over iEEG and MRI findings. IEEG findings prevailed over MRI findings.

For each selected paper, we indicated the proportions of patients falling into each of the above categories. For our meta‐analysis, we considered the lowest reliability of the reference standard for each paper. For example, if a paper included a portion of patients in which the confidence in the EZ was considered very high and another portion in which the confidence in the EZ was considered high, the overall reliability of the reference standard for this paper was rated as high.

### 2.3 Overall summary of evidence

The summary of evidence was eventually assessed using the GRADE system, according to the following categories of the level of evidence:

Very low reliability: The true effect is probably markedly different from the estimated effect.

Low reliability: The true effect might be markedly different from the estimated effect.

Moderate reliability: The authors believe that the true effect is probably close to the estimated effect.

High reliability. The authors have a lot of confidence that the true effect is similar to the estimated effect.

### 2.4 Statistical analysis

We conducted a two‐step meta‐analysis for all semiologies assessed in at least two studies. The first meta‐analysis examined the odds of occurrence of a specific ictal sign or symptom. The second meta‐analysis assessed the diagnostic accuracy of the presence or absence of specific semiological features in distinguishing lateral from mesial TLE. A sensitivity analysis was performed only including studies in which more than 50% of patients unequivocally had seizure onset in the lateral temporal lobe. Summary estimates of the odds, sensitivities, and specificities were obtained by employing a random‐effects meta‐analysis model with the inverse variance method.^9^ Between‐study heterogeneity was assessed using the Cochran's test for heterogeneity.^10^ The conventional criterion *p* < .05 for assessing statistical significance was applied. All analyses have been conducted using the statistical software R version 4.3.2, in particular the function “metaprop” within the package “meta.”^11^

## 3 RESULTS

### 3.1 Literature search

**FIGURE 1:**
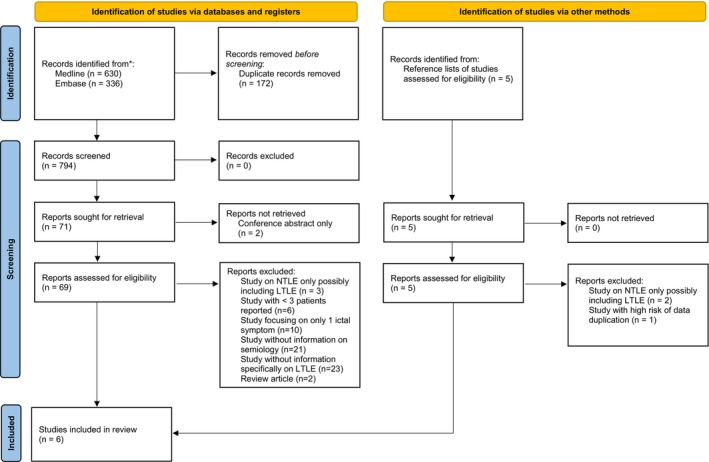
Prisma flow diagram.

The PRISMA flow diagram (Figure 1) shows that, of 794 relevant citations found, 74 articles were reviewed in full text; this includes five studies that were identified through the reference list of screened full texts.^5^, ^12^, ^13^, ^14^, ^15^ Finally, six studies fulfilled criteria for inclusion in the evidence synthesis.^4^, ^5^, ^6^, ^7^, ^8^, ^12^

Several studies of note were excluded in our screening process. One study was excluded because we considered a high risk of data duplication.^13^ The study population seemed to overlap with another study by the same working group.^8^ Five studies were not included in this review; although they dealt with neocortical TLE, there was no clear evidence that they included cases with actual lateral TLE at all.^14^, ^15^, ^16^, ^17^, ^18^

### 3.2 Included studies

All six studies that fulfilled our inclusion criteria included at least some patients with TLE confined to other neocortical temporal structures.^4^, ^5^, ^6^, ^7^, ^8^, ^12^ One study included a single patient with other neocortical TLE,^12^ the other five studies had a higher or unknown number of patients with neocortical TLE other than from actual lateral regions. No study conducted a separate analysis of the semiologies of lateral versus other neocortical temporal lobe seizures.

The basic data of the included studies are summarized in Table 1 (presented at a study‐specific level) and Table 2 (summarized data). Each study included 8–22 patients with an epileptogenic zone in the neocortical temporal lobe; the total number was 94 patients. Due to insufficient information provided in one study,^7^ the exact number of patients with unambiguous lateral TLE cannot be precisely determined, but it is calculated to be between 56 and 69 patients. In 71.2% of patients, brain MRI demonstrated a potentially epileptogenic lesion, and 42.5% of patients had EEG recordings with depth electrodes. In 97.8% of patients, resection of the presumed seizure focus was performed. In five out of six studies, information was sufficient to estimate confidence in the EZ,^5^, ^6^, ^7^, ^8^, ^12^ which was considered high or very high in 86.2% of patients.

**TABLE 1:** Description of selected studies.

Study	Number of patients with target EZ location	Number of patients with other neocortical TLE	Adult (A) Children (P)	% MRI positive	% iEEG	% operated	% Engel class I ≥ 1 year	Confidence in the EZ (% very high/High/moderate/low)
Lee, 2003	22	3	0 P/22 A	0	100	100	Unclear	0/100/0/0
Gil‐Nagel, 1997	19	1	Some P, exact number unclear	100	42.1	100	100	0/100/0/0
Maizuliana, 2020	17	9	2 P/15 A	100	23.5	100	100	82/18/0/0
O'Brien, 1996	15	Unclear	Some P, exact number unclear	100	0	100	6	60/0/0/40
Dupont, 1999	13	8	Unclear	61.5	46.2	84.6	Unclear	Unclear (low–high)
Foldvarv, 1997	8	3	Unclear	100	0	100	100	0/100/0/0

Abbreviations: A, adult patients; EZ, epileptogenic zone; iEEG, intracranial EEG; MRI, magnetic resonance imaging; P, pediatric patients; TLE, temporal lobe epilepsy.

**TABLE 2:** Summary of selected studies.

	Number of patients	% adults	% MRI positive	% iEEG	% operated	% class I ≥1 year	% high or very high confidence in the EZ
Min.	8	Unclear^a^	0	0	84.6	Unclear^a^	Unclear^b^
Max.	22	Unclear^a^	100	100	100	100%	100
Median	16	Unclear^a^	100	32.8	100	Unclear^a^	100
Total	94	Unclear^a^	71.2	42.5	97.8	Unclear^a^	86.2^b^

Abbreviations: iEEG, intracranial EEG; MRI, magnetic resonance imaging.

^a^ Insufficient data in two of six studies.

^b^ Insufficient data in one of six studies.

### 3.3 Anatomo‐clinical correlations

The ictal semiologies identified in the included studies are shown in Table 3. In lateral TLE, the most commonly observed semiologies comprised oral automatisms, manual automatisms, and behavioral arrests. Only one study examined whether these semiologies occurred at the onset of seizures or during propagation.^7^ The wide latency range (0–112 s after seizure onset) indicates that oral and manual automatisms as well as behavioral arrest mainly emerge during seizure propagation.

**TABLE 3:** Anatomo‐clinical correlations.

Ictal sign or symptom	Assessed in #studies (S) #patients (P)	% reported (min–max)	More at onset or during propagation	Overall grade level (high, moderate, low) that the sign/symptom is associated with the brain region
Non‐lateralizing somatic aura	2 S/36 P	5.9–10.5	Not reported	Low
Epigastric aura	4 S/66 P	0–13.6	Not reported	Low
Olfactory/gustatory aura	3 S/42 P	0–0	N/a	Low
Dizziness/cephalic aura	3 S/47 P	11.8–50	Not reported	Low
Fear aura/psychic aura	5 S/81 P	0–40	Not reported	Low
Auditory aura	3 S/54 P	5.9–9.1	Not reported	Low
Visual aura	3 S/54 P	0–6.7	Not reported	Low
Autonomic aura	3 S/40 P	20–37.5	Not reported	Low
Emotional aura	1 S/8 P	12.5	Not reported	Low
Sensory aura	1 S/15 P	6.7	Not reported	Low
Contralateral dystonic posturing	3 S/45 P	0–60	1 S: onset 26 s^a^	Low
Ipsilateral dystonic posturing	1 S/13 P	23.1	Not reported	Low
Oral automatisms	3 S/51 P	29.4–73.3	1 S: onset 11 s^a^	Low
Manual automatism	5 S/86 P	15.4–80	1 S: onset 14 s^a^	Low
Versive seizure	2 S/39 P	0–13.6	Not reported	Low
Head turning	1 S/15 P	60	1 S: onset 14 s^a^	Low
(sudden) GTCS	3 S/54 P	5.3–33.3	1 S: onset 12 s^a^	Low
Face clonic/grimace	3 S/51 P	11.8–33.3	1 S: onset 19 s^a^	Low
Nose wiping	1 S/17 P	11.8	Not reported	Low
Restlessness	1 S/15 P	46.77	1 S: onset 8 s^a^	Low
Arrest	3 S/56 P	13.6–86.7	Not reported	Low
Speech disturbance	3 S/51 P	5.3–20	Not reported	Low
Drooling	1 S/17 P	5.9	Not reported	Low
Post‐ictal aphasia	2 S/32 P	5.9–20	Not reported	Low
Headache	1 S/22 P	4.5	Not reported	Low
Experiential aura	1 S/19 P	47.3	Not reported	Low

Abbreviations: GTCS, generalized tonic–clonic seizure; P, patients; s, second; S, studies.

^a^ Median.

**FIGURE 2:**
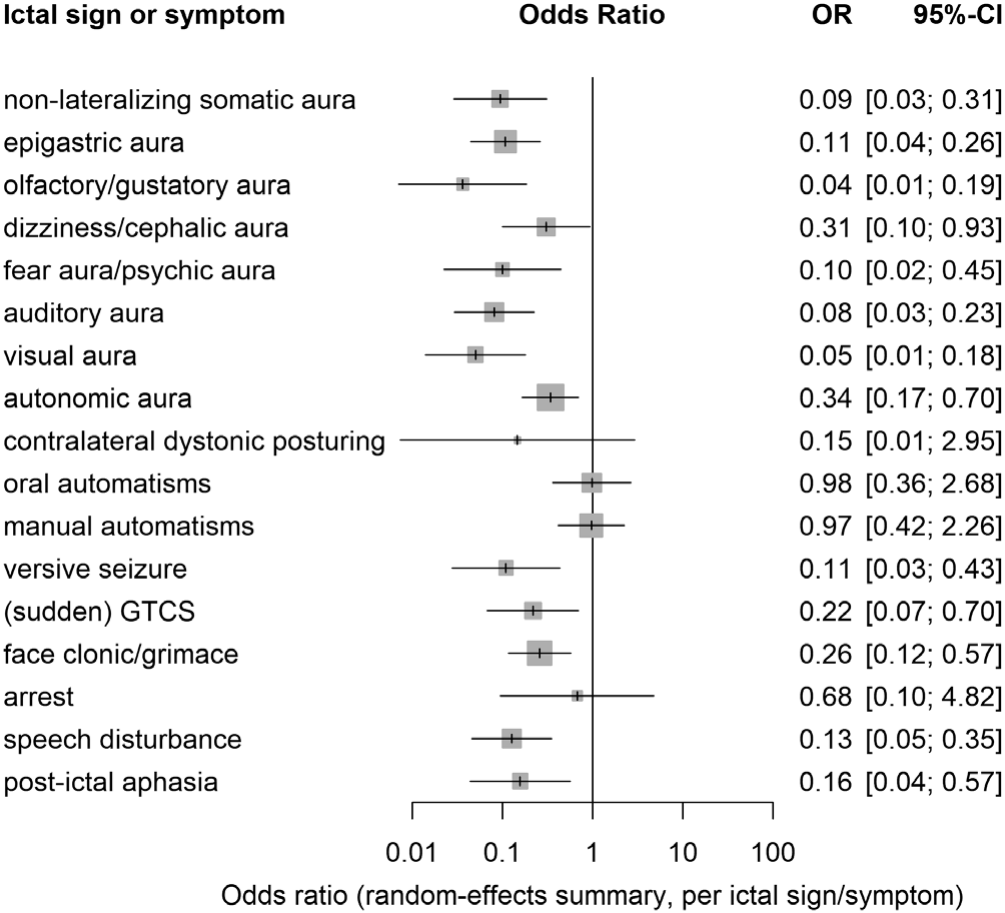
Meta‐analysis of ictal signs and symptoms in lateral temporal lobe epilepsy. This forest plot shows random‐effects summary odds ratios (ORs) for the occurrence of specific ictal signs or symptoms in patients with lateral TLE. An OR >1 indicates that the sign or symptom is more likely to be present than absent, whereas an OR <1 indicates that the sign or symptom is more likely to be absent than present. 95% CI, 95% confidence interval; GCTS, generalized tonic–clonic seizure; OR, odds ratio.

The results of our meta‐analysis on the odds of an ictal sign or symptom occuring in lateral TLE are presented in Table 4 and Figure 2; the study‐specific odds ratios are provided in the Figure S1. No semiological feature had an overall odds suggestive of an association with lateral TLE. The same was true for our sensitivity analysis that only included the three studies in which more than 50% of patients unequivocally had seizure onset in the lateral temporal lobe (Table S1, Figure S2). Overall, according to our GRADE evaluation, there is very low evidence that any signs or symptoms assessed are associated with localization of the EZ. This assessment was largely driven by substantial imprecision, as reflected in the wide confidence intervals in our meta‐analysis, and by potential confounding due to the inclusion of other neocortical TLE cases in the included studies.

**TABLE 4:** Meta‐analysis on the odds of occurrence of an ictal sign or symptom in lateral TLE.

Ictal sign or symptom	Assessed in #studies (S) #patients (P)	Odds overall	Heterogeneity
Non‐lateralizing somatic aura	2 S/36 P	.09 [95% CI .03–.31]	*p* = .6194
Epigastric aura	4 S/66 P	.11 [95% CI .04–.26]	*p* = .6299
Olfactory/gustatory aura	3 S/42 P	.04 [95% CI .01–.19]	*p* = .9159
Dizziness/cephalic aura	3 S/47 P	.31 [95% CI .10–.93]	*p* = .1134
Fear aura/psychic aura	5 S/81 P	.10 [95% CI .02–.45]	*p* = .0243
Auditory aura	3 S/54 P	.08 [95% CI .03–.23]	*p* = .9237
Visual aura	3 S/54 P	.05 [95% CI .01–.18]	*p* = .8723
Autonomic aura	3 S/40 P	.34 [95% CI .17–.7]	*p* = .6498
Contralateral dystonic posturing	3 S/45 P	.15 [95% CI .01–2.95]	*p* = .0037
Oral automatisms	3 S/51 P	.98 [95% CI .36–2.68]	*p* = .0571
Manual automatisms	5 S/86 P	.97 [95% CI .42–2.26]	*p* = .0142
Versive seizure	2 S/39 P	.11 [95% CI .03–.43]	*p* = .2741
(sudden) GTCS	3 S/54 P	.22 [95% CI .07–.70]	*p* = .1184
Face clonic/grimace	3 S/51 P	.26 [95% CI .12–.57]	*p* = .2896
Arrest	3 S/56 P	.68 [95% CI .10–4.82]	*p* = .0005
Speech disturbance	3 S/51 P	.13 [95% CI .05–.35]	*p* = .3305
Post‐ictal aphasia	2 S/32 P	.16 [95% CI .04–.57]	*p* = .2543

*Note*: Odds overall = summary estimate of the odds, resulting from the meta‐analysis. Values >1 indicate that the occurrence of an ictal sign or symptom is more likely than the absence of an ictal sign or symptom in lateral TLE. Values <1 indicate that the absence of an ictal sign or symptom is more likely than the occurrence of an ictal sign or symptom in lateral TLE. Test for heterogeneity: if *p* < .05→study‐specific estimates of the odds are heterogeneous, so the overall summary estimate has to be interpreted with some caution.

Abbreviations: CI, confidence interval; GTCS, generalized tonic–clonic seizure; P, patients; S, studies.

### 3.4 Comparison of lateral and mesial TLE

Five out of six studies included in this review compared ictal signs and symptoms in lateral to those in mesial TLE.^4^, ^5^, ^6^, ^7^, ^12^ The comparative findings are given in Table 5. In lateral compared to mesial temporal lobe seizures, there was a notably lower prevalence of epigastric and olfactory/gustatory auras.

**TABLE 5:** Comparison of lateral to mesial TLE.

Ictal sign or symptom	Assessed in #studies (S) #patients (P) lateral TLE/# patients mesial TLE	Lateral TLE % reported (min–max)	Mesial TLE % reported (min–max)
Experiential aura	1 S/19/16 P	47.3	0
Non‐lateralizing somatic aura	2 S/36/36 P	5.9–10.5	0–18.8
Epigastric aura	3 S/44/56 P	0–12.5	31.3–40
Olfactory/gustatory aura	3 S/42/67 P	0	0–18.8
Fear aura/psychic aura	4 S/58/87 P	0–40	18.8–35.5
Dizziness/cephalic aura	2 S/25/40 P	11.8–50	0–40
Auditory aura	2 S/32/51 P	5.9–6.7	0–12.9
Visual aura	2 S/32/51 P	0–6.7	0–5
Autonomic aura	3 S/40/71 P	20–37.5	5–48.4
Emotional aura	1 S/8/20 P	12.5	15
Sensory aura	1 S/15/31 P	6.7	16.1
Contralateral dystonic posturing	3 S/45/96 P	0–60	40–74.2
Ipsilateral dystonic posturing	1 S/13/45 P	23.1	2.2
Oral automatisms	3 S/51/67 P	29.4–73.3	31.3–80.6
Manual automatisms	4 S/64/112 P	15.4–80	25–83.9
Versive seizure	1 S/17/20 P	0	0
Head turning	1 S/15/31 P	60	64.5
(sudden) GTCS	2 S/32/51 P	17.6–33.3	10–41.9
Face clonic/grimace	3 S/51/67 P	11.8–33.3	15–68.8
Nose wiping	1 S/17/20 P	11.8	30
Restlessness	1 S/15/31 P	46.7	45.2
Arrest	2 S/34/47 P	26.3–86.7	37.5–93.5
Speech disturbance	3 S/51/67 P	5.3–20	15–22.5
Drooling	1 S/17 P	5.9	20
Post‐ictal aphasia	2 S/32 P	5.9–20	12.9–30

Abbreviations: GTCS, generalized tonic–clonic seizure; P, patients; S, studies; TLE, temporal lobe epilepsy.

The results of a meta‐analysis of the diagnostic accuracy for differentiating seizures originating in lateral from those in mesial temporal lobe structures with regard to the presence or absence of relevant ictal signs and symptoms are listed in Table 6; study‐specific diagnostic odds ratios are provided in the Figure S2. The presence of auditory auras was highly specific for distinguishing lateral from mesial TLE (specificity .98 [95% CI .88–1.00]) with an overall sensitivity of .06 [95% CI .02–.22].

**TABLE 6:** Meta‐analysis on the diagnostic accuracy for differentiating lateral from mesial TLE with regard to the presence or absence of relevant semiological features.

	Overall sensitivity	Heterogeneity	Overall specificity	Heterogeneity
Oral automatism (3 studies)	.50 [95% CI .27–.73]	p = .0571	.21 [95% CI .13–.32]	*p* = .8975
Auditory aura (2 studies)	.06 [95% CI .02–.22]	*p* = .9272	.98 [95% CI .88–1.00]	*p* = .8316
Lack of dystonic posturing (3 studies)	.87 [95% CI .25–.99]	p = .0037	.55 [95% CI .35–.73]	*p* = .0339
Lack of epigastric aura (3 studies)	.93 [95% CI .80–.98]	*p* = .6223	.38 [95% CI .26–.51]	*p* = .8306
Lack of olfactory/gustatory aura (3 studies)	.96 [95% CI .84–.99]	p = .9159	.13 [95% CI .06–.25]	*p* = .3568
Manual automatisms (4 studies)	.45 [95% CI .22–.70]	*p* = .0154	.35 [95% CI .17–.60]	*p* = .0022
Arrest (2 studies)	.59 [95% CI .08–.96]	*p* = .0016	.32 [95% CI .01–.95]	*p* < .0001

*Note*: Some studies did not include patients with mesial TLE; therefore, the number of studies differs from the other tables.

Abbreviations: CI, confidence interval; TLE, temporal lobe epilepsy.

Lack of epigastric or olfactory/gustatory aura was associated with a high sensitivity for differentiating lateral from mesial TLE (.93 [95% CI .80–.98] and .96 [95% CI .84–.99], respectively); specificity was .38 [95% CI .26–.51] for lack of epigastric aura; and .13 [95% CI .06–.25] for lack of olfactory/gustatory aura. For manual automatisms and arrest reaction, heterogeneity was very high, limiting the interpretability of pooled effect estimates.

## 4 DISCUSSION

Our systematic review and meta‐analysis yield three main findings: First, the lateral temporal lobe lacks clearly defined anatomical boundaries, and semiological studies rarely examine this region independently. Second, the semiology of lateral temporal lobe seizures is heterogeneous; auditory auras represent the only specific semiology for lateral TLE, though they occur only in a minority of cases. Third, despite this heterogeneity, certain semiological features may aid in distinguishing lateral from mesial temporal lobe seizures. Notably, the absence of specific symptoms such as epigastric or olfactory/gustatory aura may be more indicative for lateral TLE than the presence of particular ones.

### 4.1 Defining boundaries of the lateral temporal lobe

Similar to what has been demonstrated in systematic reviews of neighboring brain regions, the anatomical delineation of the lateral temporal lobe is imprecise.^19^, ^20^, ^21^

All studies included in this systematic review did not exclusively focus on lateral TLE but also included other neocortical TLE, that is, seizures from temporo‐basal and temporal‐polar structures. It is likely that this inaccuracy is a confounder for the current findings.

A precise anatomo‐clinical definition of the lateral temporal lobe could help standardize future studies and improve comparability of findings. In the long term, this may also contribute to better surgical outcomes in epilepsy patients undergoing resection of the lateral temporal lobe, which currently shows less favorable prognoses compared to mesial TLE.^22^, ^23^

### 4.2 Semiology of lateral temporal lobe seizures

In our systematic review, the semiology of lateral temporal lobe seizures was highly heterogeneous: Based on data from 94 patients, 24 distinct ictal phenomena could be identified.

Auditory auras have traditionally been considered as the most prominent symptom of lateral temporal lobe seizures.^2^, ^24^, ^25^ In our analysis, auditory auras were present in only 5–10% of patients with lateral TLE. In more general epilepsy populations, the prevalence of auditory auras ranges from 1.5 to 6%,^26^, ^27^ making the rate of auditory auras in the studies included in our systematic review surprisingly low.

More commonly seen semiologies in this analysis were oral and manual automatisms as well as arrest reactions. In our meta‐analysis, only manual automatisms had an overall odds suggestive of an association with lateral TLE, although this association was not statistically significant. Automatisms are considered a common sign of temporal lobe epilepsy;^2^, ^28^, ^29^, ^30^ however, this is also seen in seizures originating from other brain regions like the frontal,^31^ parietal,^32^ and occipital lobe,^33^ indicating that it commonly does not manifest at seizure onset but during propagation.

### 4.3 Differentiating ictal semiologies in lateral from mesial temporal lobe epilepsy

Although the association of individual semiological features with lateral TLE was generally weak, our meta‐analysis demonstrated the potential value of specific semiological features in distinguishing lateral from mesial TLE.

Auditory auras were notably associated with a very high specificity for distinguishing lateral from mesial TLE. In previous analyses, auditory auras have been found to be a reliable predictor for lateralization of the epileptogenic zone to the dominant hemisphere.^26^ Our results show that they can also be a reliable predictor of lateral as opposed to mesial TLE. However, the occurrence of auditory auras was also very rare in lateral TLE, resulting in an overall sensitivity of only 6%.

Indeed, lateral TLE appears to be characterized more by the absence than by the presence of certain semiological features. Notably, the absence of epigastric auras as well as the lack of olfactory/gustatory auras was associated with high sensitivity for lateral when compared to mesial TLE. However, the specificity of these findings ranged from low to moderate, which may constrain their clinical utility. Nevertheless, Ebner has already found in 1994 the significance of aura types in differentiating lateral from mesial TLE.^34^ Furthermore, it has already been suggested in the past to consider lateral TLE particularly when temporal epileptiform discharges are found in EEG and when typical semiological features of mesial TLE are absent.^24^, ^35^ The findings of our meta‐analysis support the latter part of this suggestion. Stereoelectroencephalography was used in less than 50% of patients in the six included studies but likely paves the way for assigning ictal clinical signs and symptoms to clearly defined subregions of the temporal lobe.^36^, ^37^

### 4.4 Strengths and limitations

The major strength of this review is its strict compliance with PRISMA guidelines and its careful appraisal of the reliability of the reference standard with regard to the epileptogenic zone in the included studies.

The main limitation of this work, namely that all included studies did not exclusively focus on the actual lateral temporal lobe but also considered patients with seizures confined to other neocortical temporal regions, has already been discussed above. All included studies had a relatively low number of patients, resulting in a total sample of only 94 patients across six studies. Furthermore, the individual epilepsy centers may have interpreted certain ictal phenomena differently and used variable nomenclature; this study heterogeneity may have blurred the results of the current systematic review. The vast majority of patients in the included studies were adults which limits the application of the current results to pediatric patients. Two of the studies included in this work were not acquired through the primary literature search but through secondary sources.^5^, ^12^ This suggests that the search strategy might have been too restrictive. However, it was important for us to adhere to the standardized framework for systematic reviews on seizure semiology within the Paros project.

## 5 CONCLUSIONS

The anatomical boundaries of the lateral temporal lobe are poorly defined in all studies considered in this systematic review. Furthermore, only about two‐thirds of patients clearly had seizure onset confined to the lateral temporal lobe. Nevertheless, some semiological features of seizures originating from this brain region have been identified, which can be particularly helpful in distinguishing lateral from mesial TLE. Foremost among these are auditory auras, which, although a very rare symptom, can be considered quite specific for lateral temporal lobe seizures. Otherwise, epigastric and even more so olfactory/gustatory auras may hint to mesial rather than to lateral TLE.

## FUNDING INFORMATION

This study was in part funded by the financial resources of the Friedrich von Bodelschwingh Endowed Professorship for Clinical and Experimental Epileptology at the Department of Neurology, Charité–Universitätsmedizin Berlin (to MH).

## CONFLICT OF INTEREST STATEMENT

Jakob Dörrrfuß reports personal fees from Eisai within the past 3 years, unrelated to the contents of the article. Georg Zimmermann does not declare any potential conflicts of interest. Martin Holtkamp has received speaker's honoraria from Angelini, Bial, Danone, Desitin, Eisai, Jazz Pharma, Neuraxpharm, and UCB within the last 3 years, unrelated to the contents of the article.

**Test yourself**

1. Which semiological feature is highly specific for seizures originating from the lateral temporal lobe?
  - olfactory auras
  - oral automatisms
  - epigastric auras
  - auditory auras
  - post‐ictal aphasia
2. Which semiological pattern is more suggestive of lateral rather than mesial temporal lobe epilepsy?
  - Presence of epigastric aura
  - Presence of olfactory or gustatory aura
  - Early manual automatisms at seizure onset
  - Absence of epigastric and olfactory/gustatory auras
  - Presence of post‐ictal confusion
3. Which diagnostic tool is most likely to improve the anatomo‐clinical delineation of the lateral temporal lobe?
  - High‐density scalp EEG.
  - Stereoelectroencephalography
  - Standard video‐EEG monitoring
  - Functional MRI during language tasks
  - PET imaging assessing interictal hypometabolism

*Answers may be found in the* *supporting information*

## Supporting information


Figure S1.

Data S1.

Appendix S1.

## Data Availability

Data sharing is not applicable to this article as no new data were created or analyzed in this study.
