# Supplementary material for: Ictal semiology in lateral temporal epilepsy: A systematic review and meta‐analysis
Source: Epileptic Disord. 2026 Feb 2;28(3):678–89. doi: 10.1002/epd2.70189 (PMC13276704; doi:10.1002/epd2.70189)
Supplement: Supplementary file 1 — Figure S1. [file EPD2-28-678-s003.docx]

**ICTAL SEMIOLOGY IN LATERAL TEMPORAL EPILEPSY: A SYSTEMATIC REVIEW AND META-ANALYSIS**

Jakob I. Doerrfuss, Georg Zimmermann, Martin Holtkamp

**Supplementary Material**

**Figure S1 - Meta-analysis on the odds of occurrence of an ictal sign or symptom in lateral TLE**

*Figure S1a : Meta-analysis on non-lateralizing somatic aura*


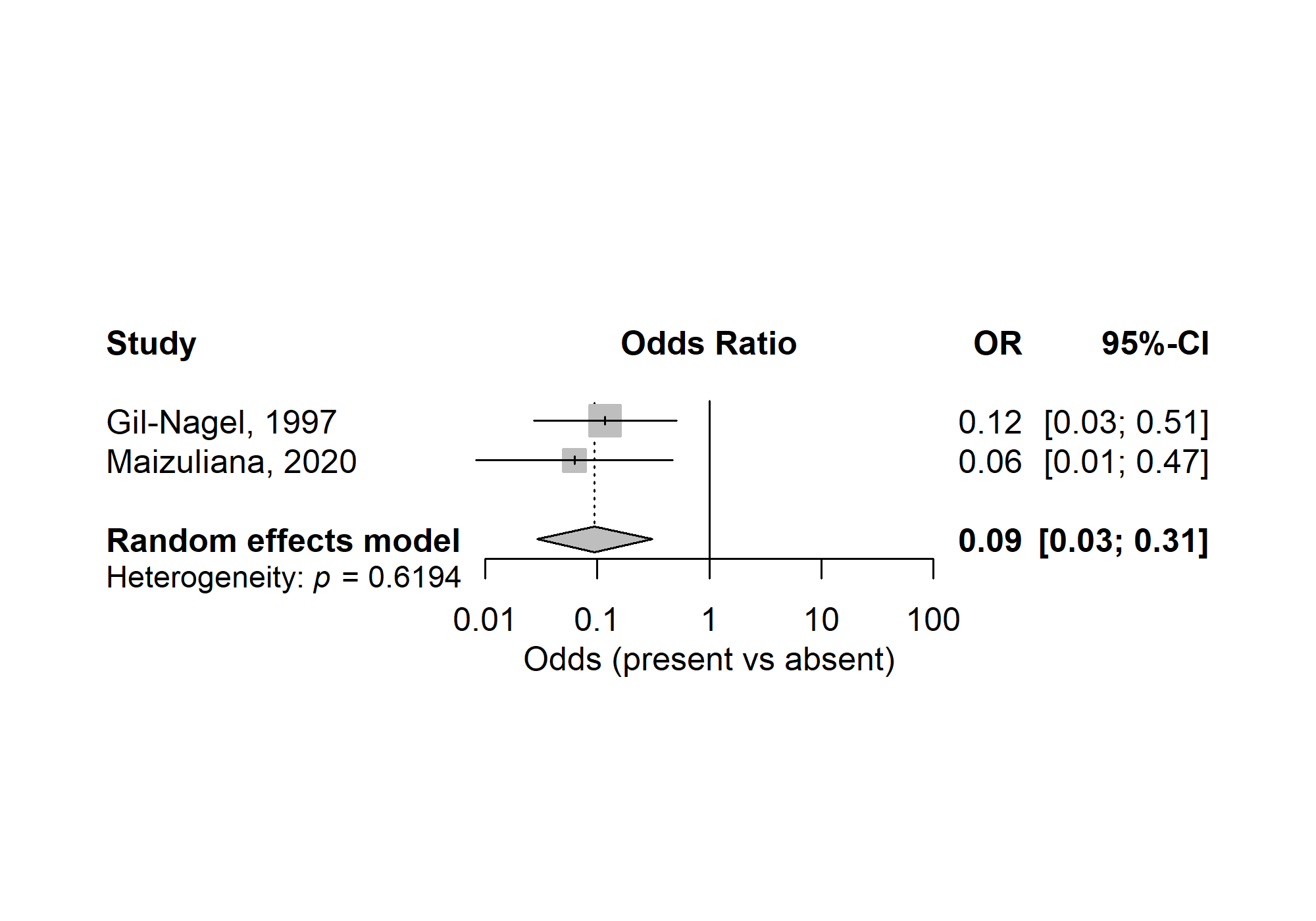


*Figure S1b : Meta-analysis on epigastric aura*

*
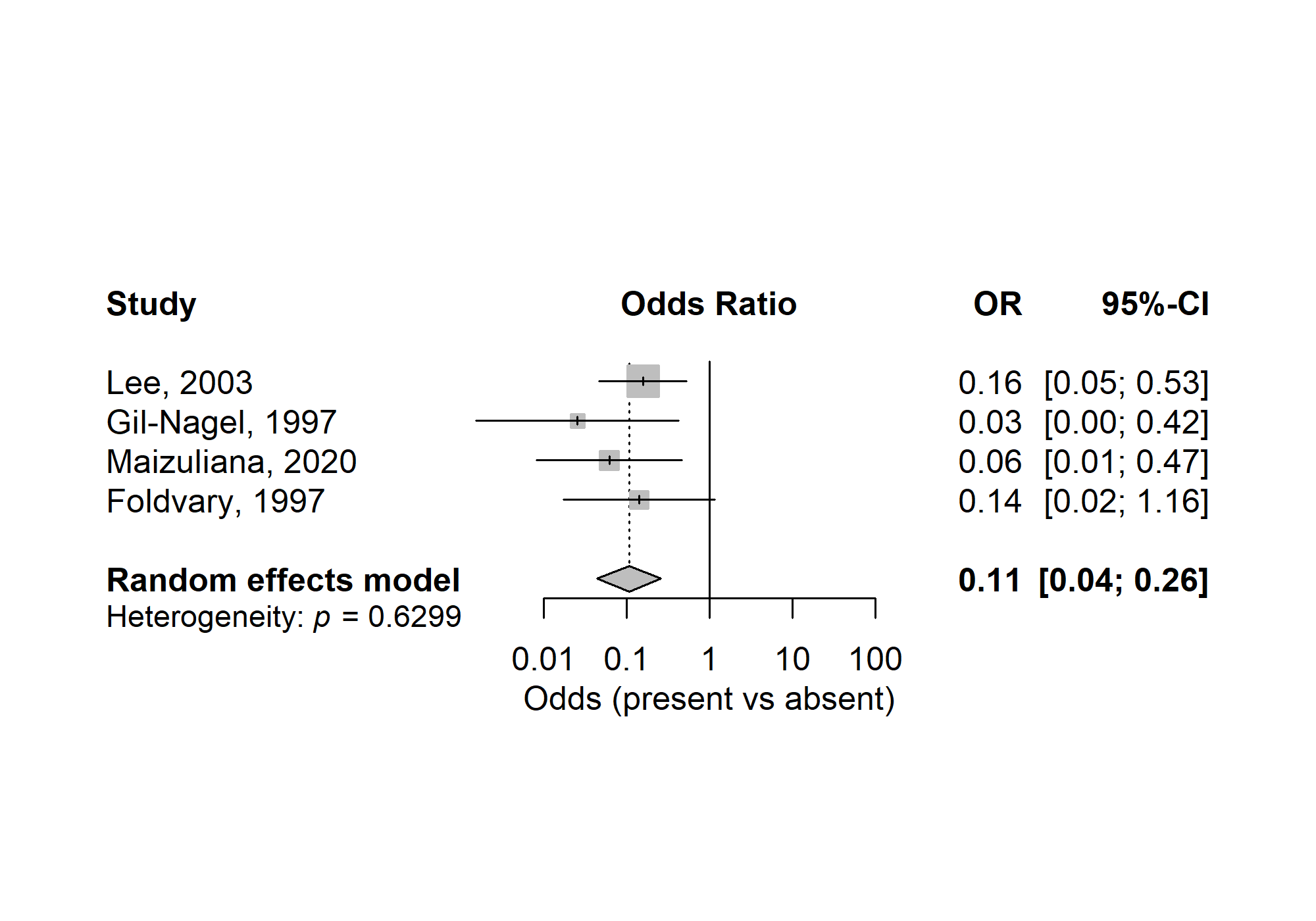
*

*Figure S1c : Meta-analysis on olfactory/gustatory aura*

*
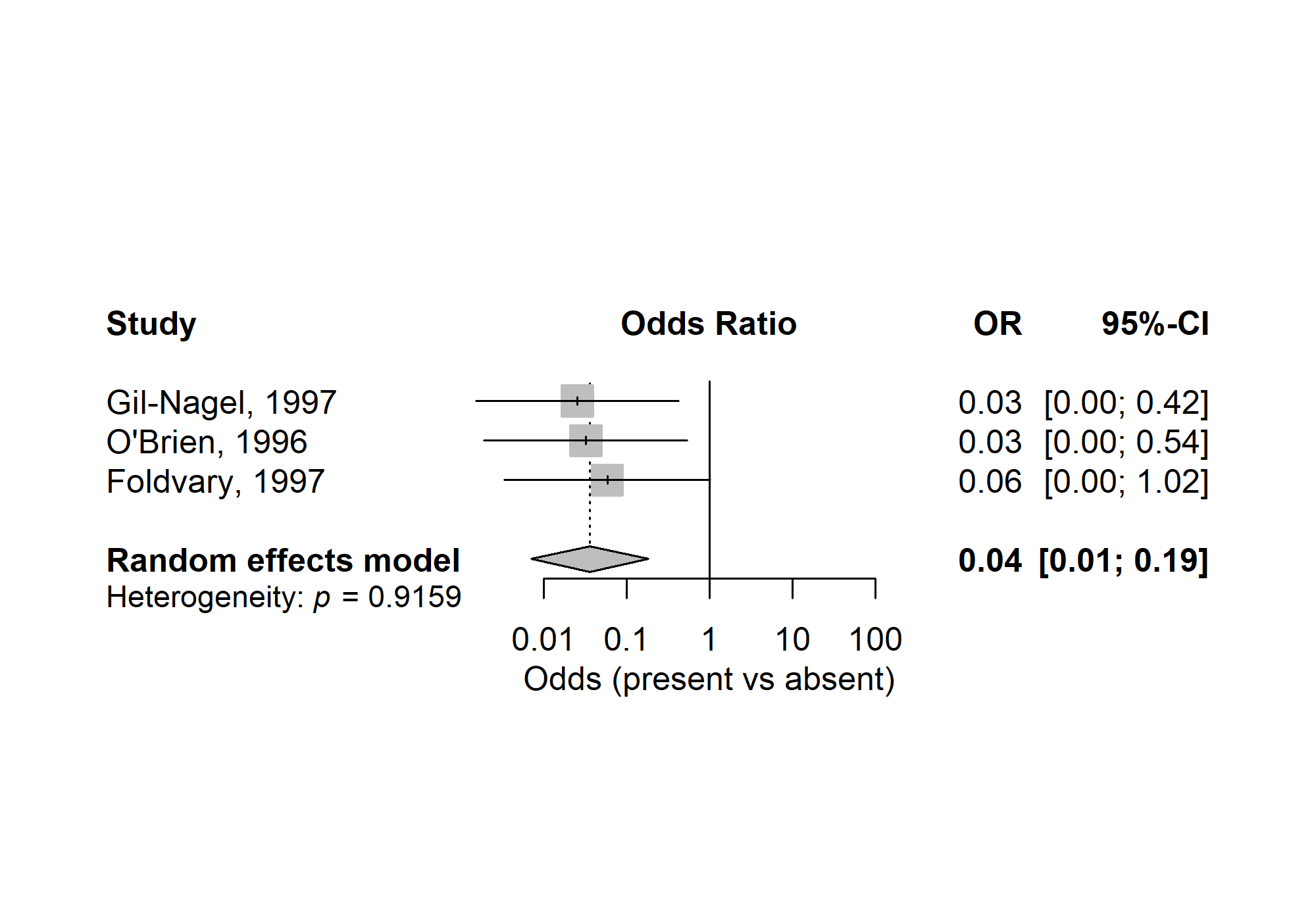
*

*Figure S1d: Meta-analysis on dizziness/cephalic aura*

*
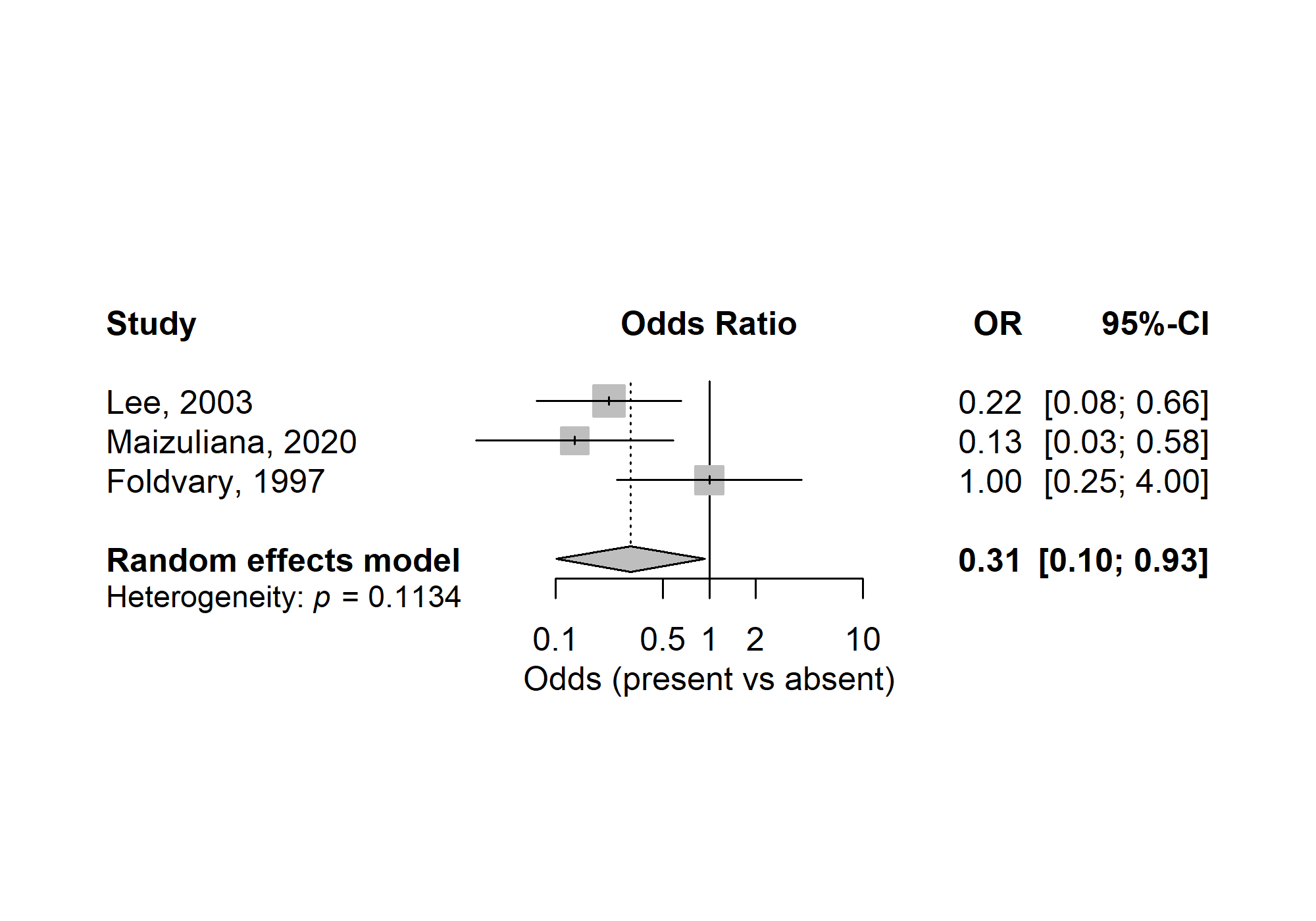
*

*Figure S1e : Meta-analysis on fear aura/psychic aura*


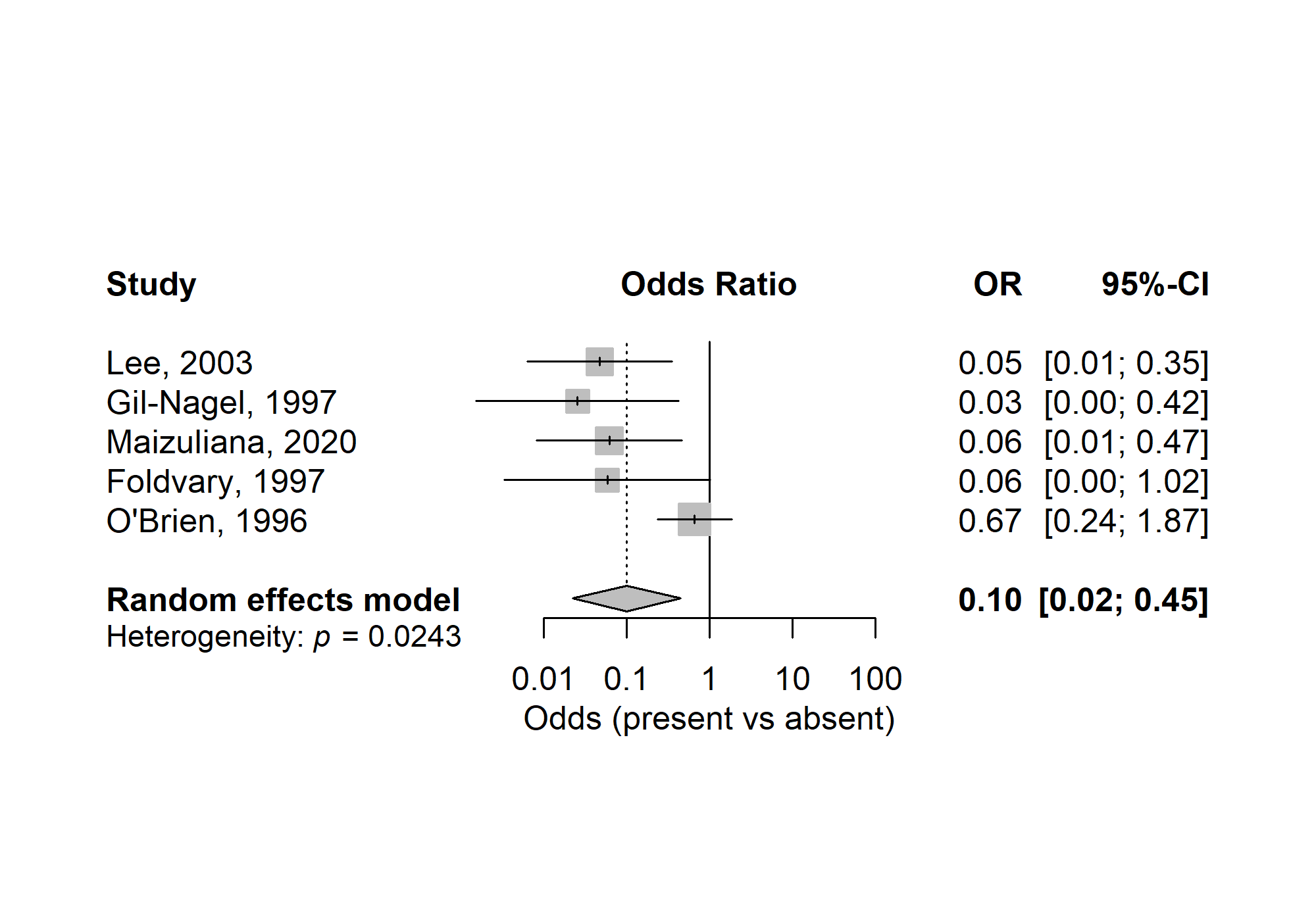


*Figure S1f : Meta-analysis on auditory aura*

**
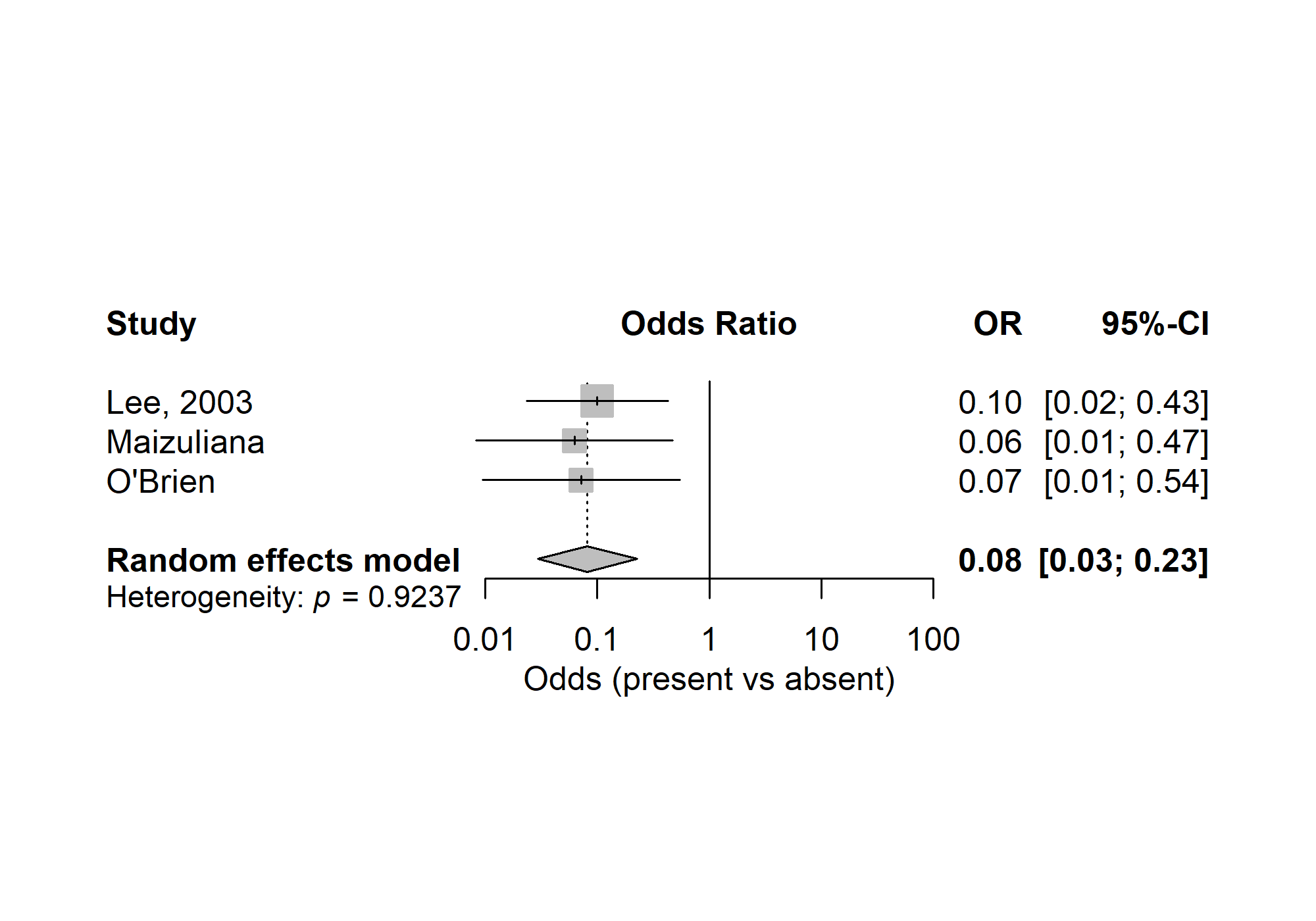
**

*Figure S1g : Meta-analysis on visual aura*

*
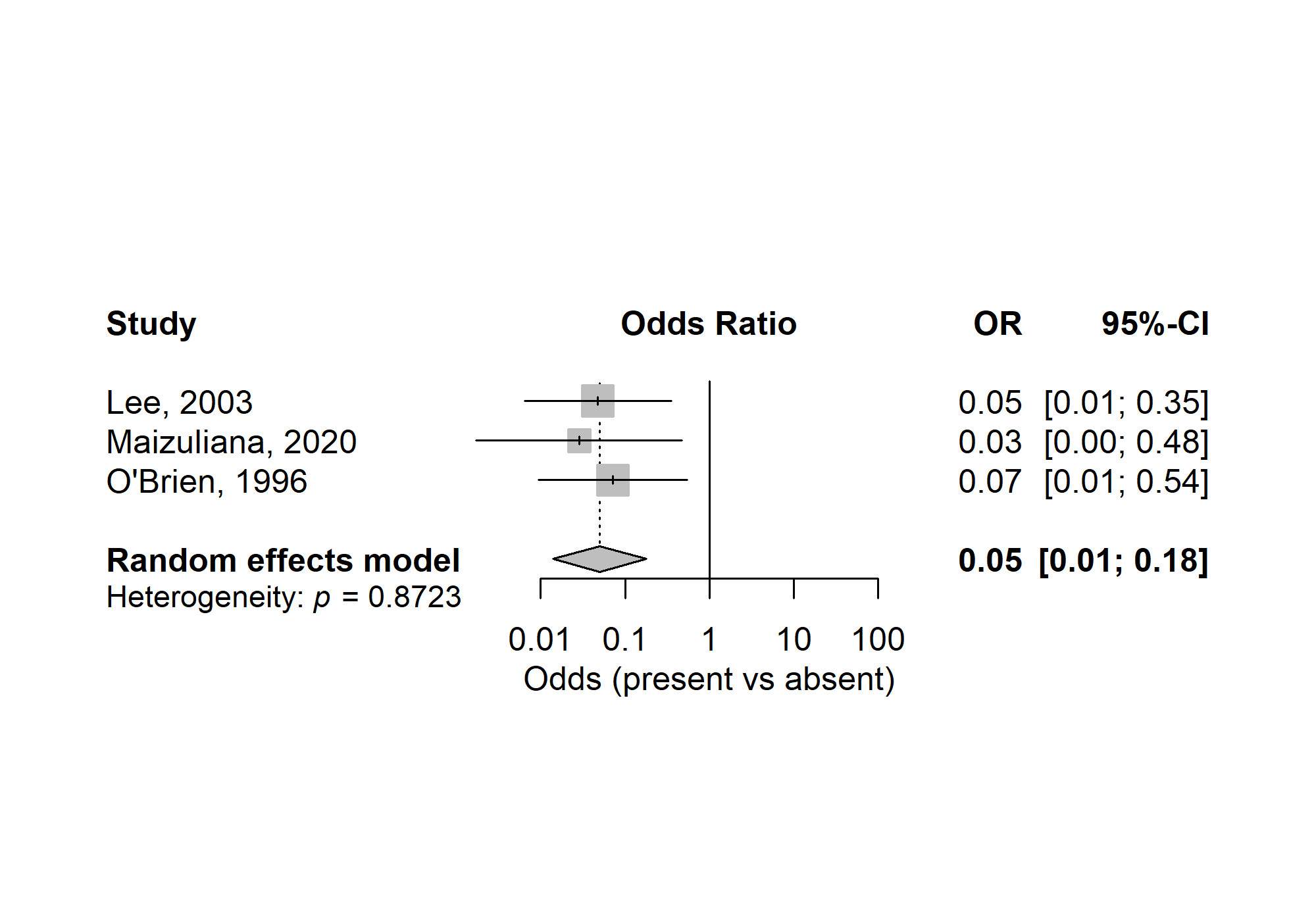
*

*Figure S1h : Meta-analysis on autonomic aura*


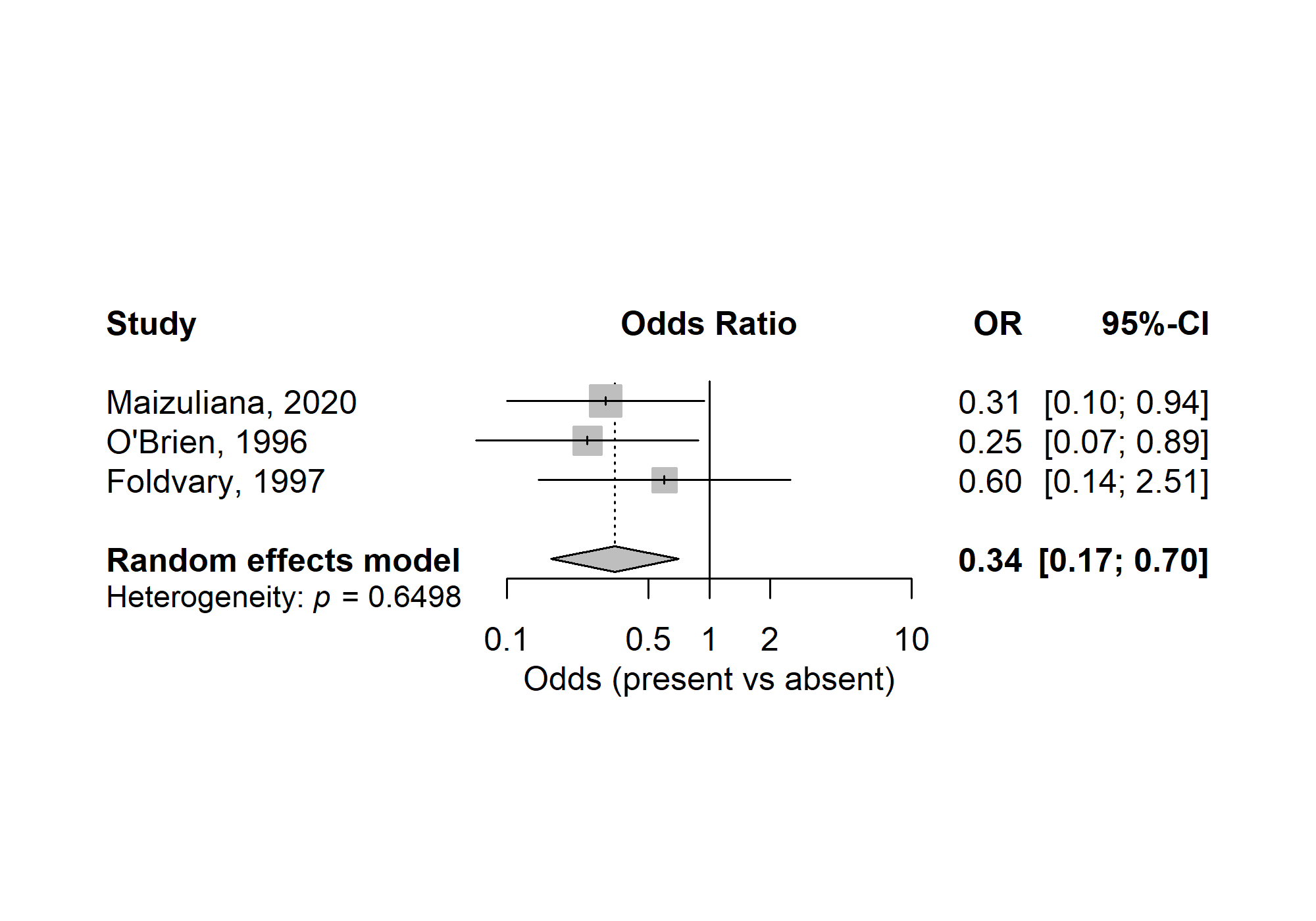


*Figure S1i : Meta-analysis on contralateral dystonic posturing*


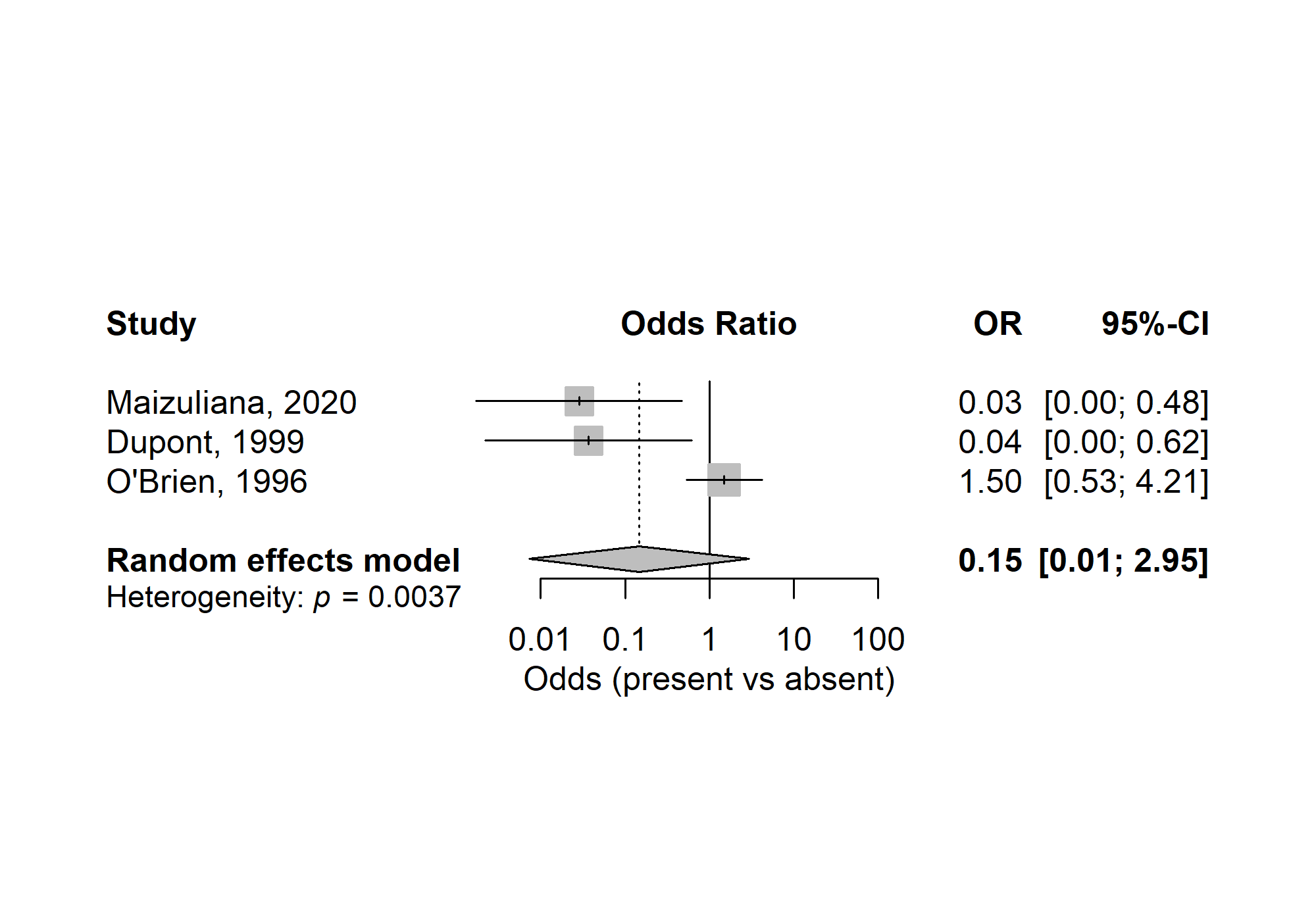


*Figure S1j : Meta-analysis on oral automatisms*


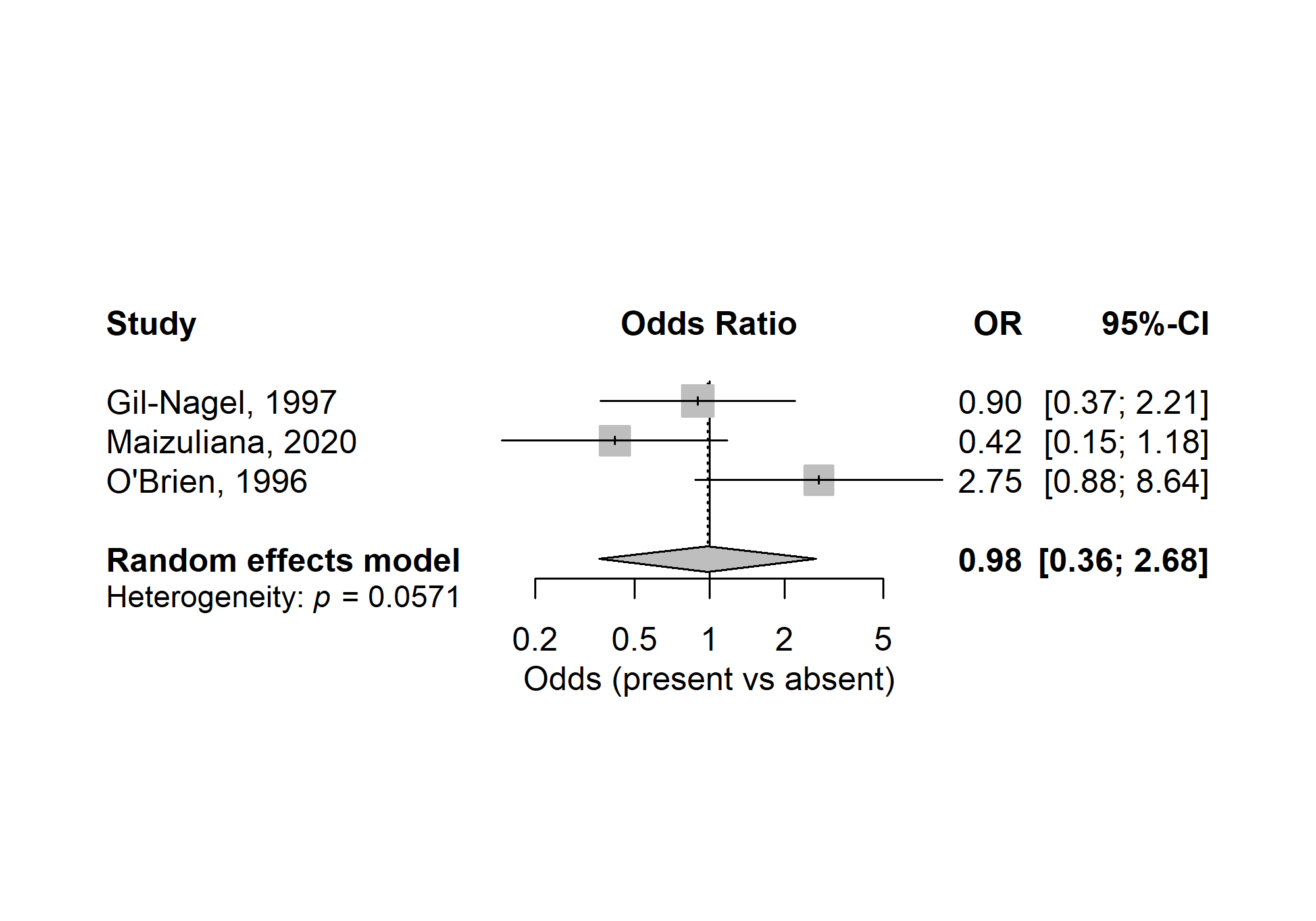


*Figure S1k : Meta-analysis on manual automatisms*


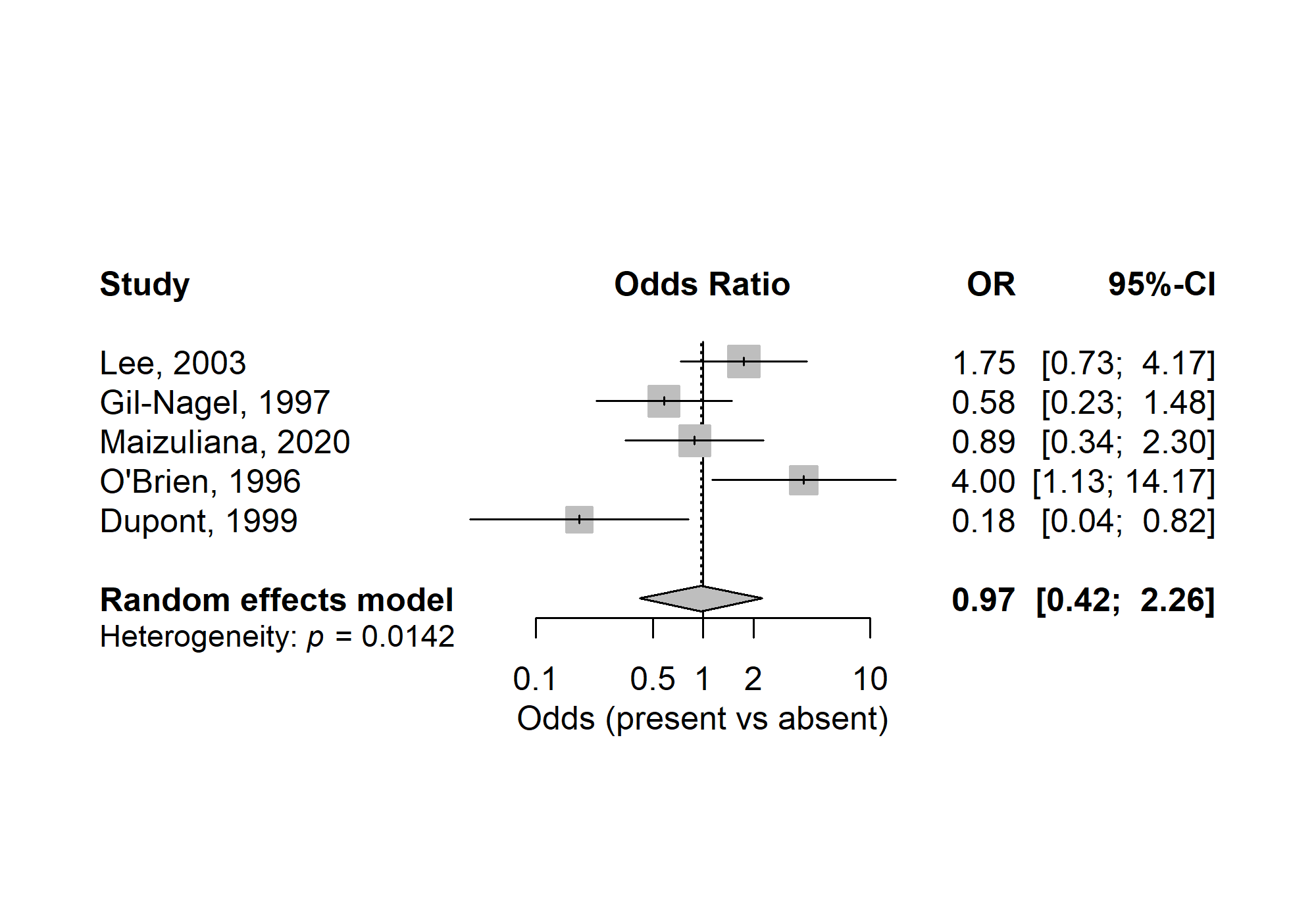


*Figure S1l : Meta-analysis on versive seizures*


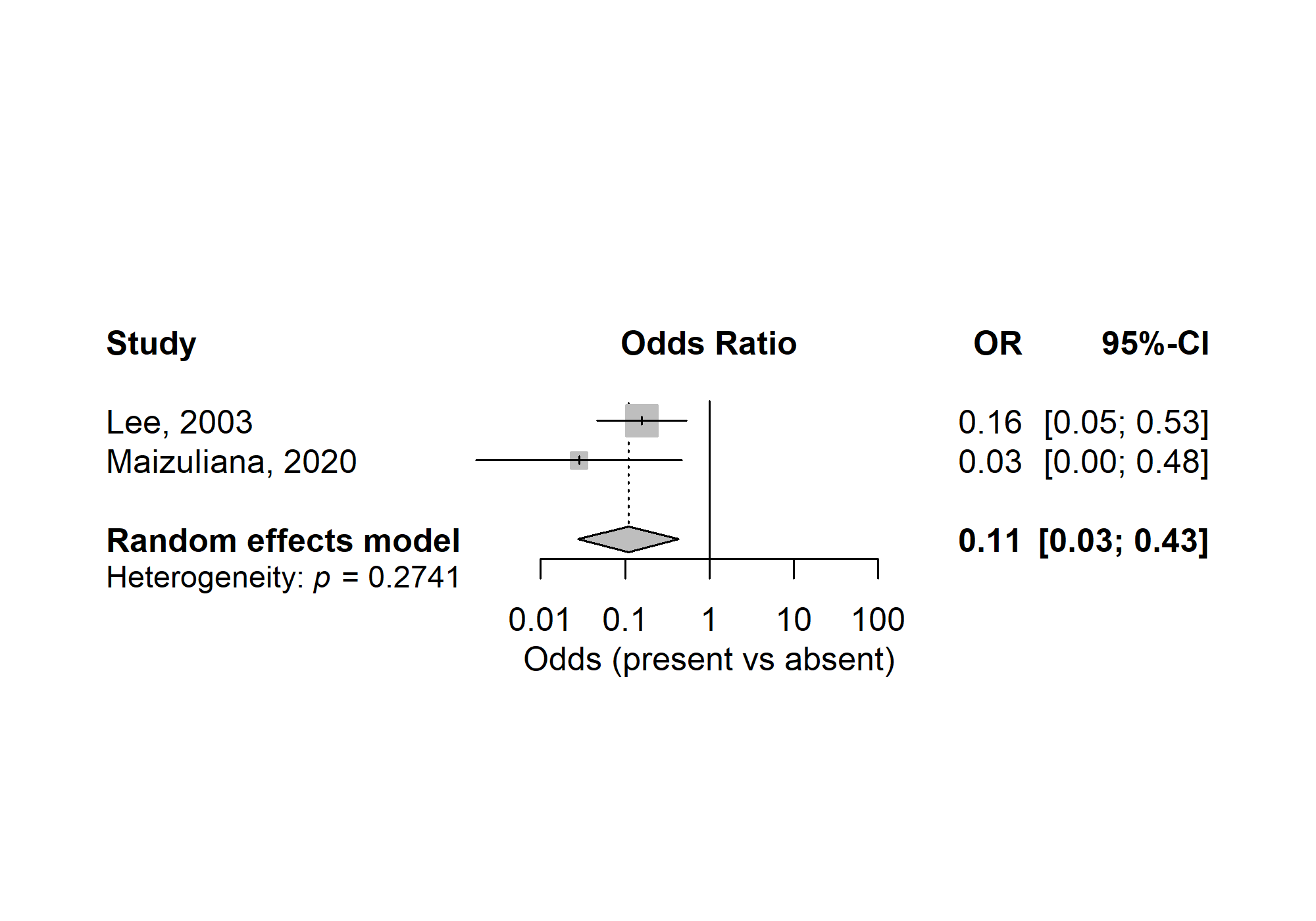


*Figure S1m: Meta-analysis on sudden generalized tonic-clonic seizures*


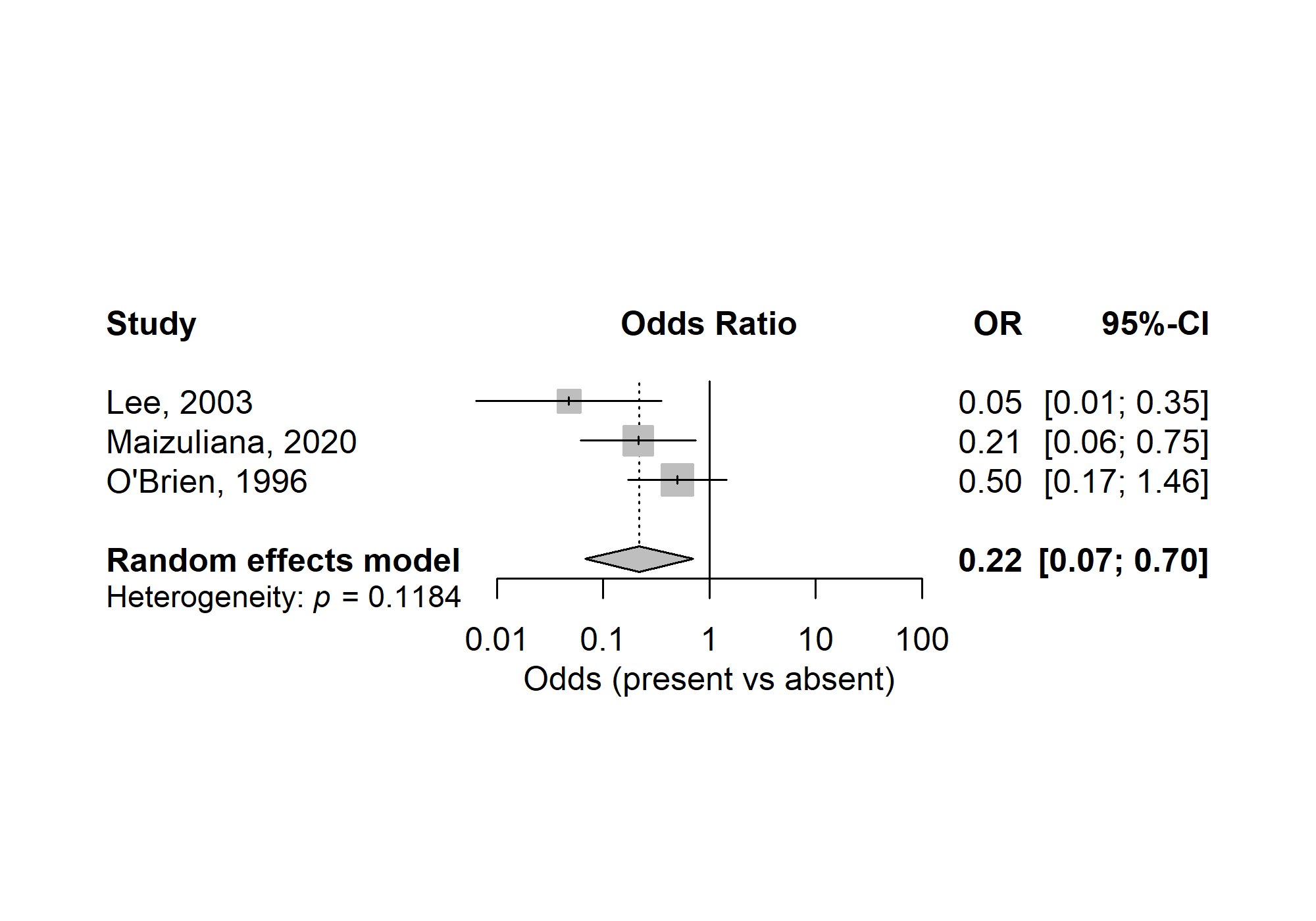


*Figure S1n : Meta-analysis on face clonic/grimace semiology*


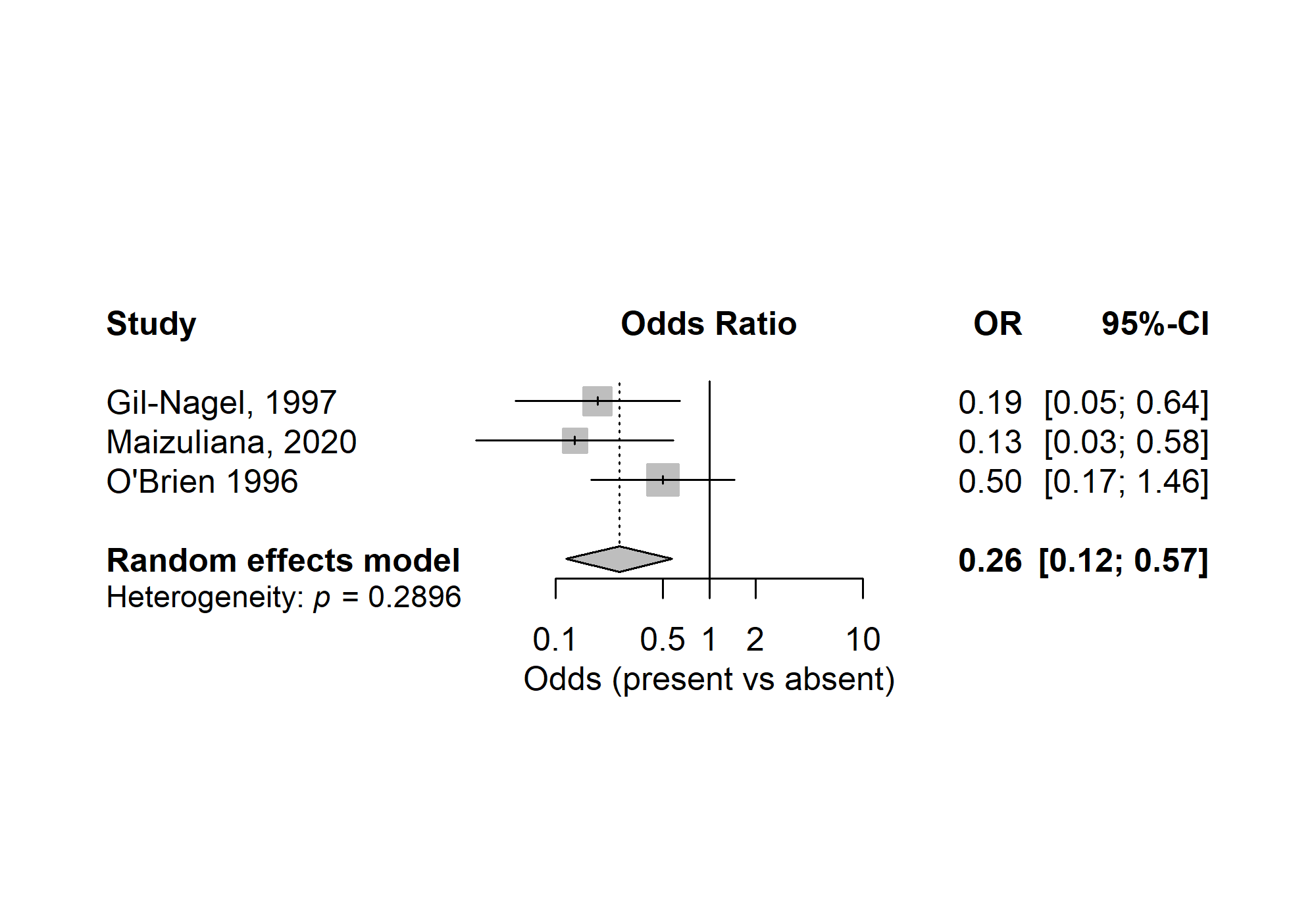


*Figure S1o: Meta-analysis on arrest reaction*


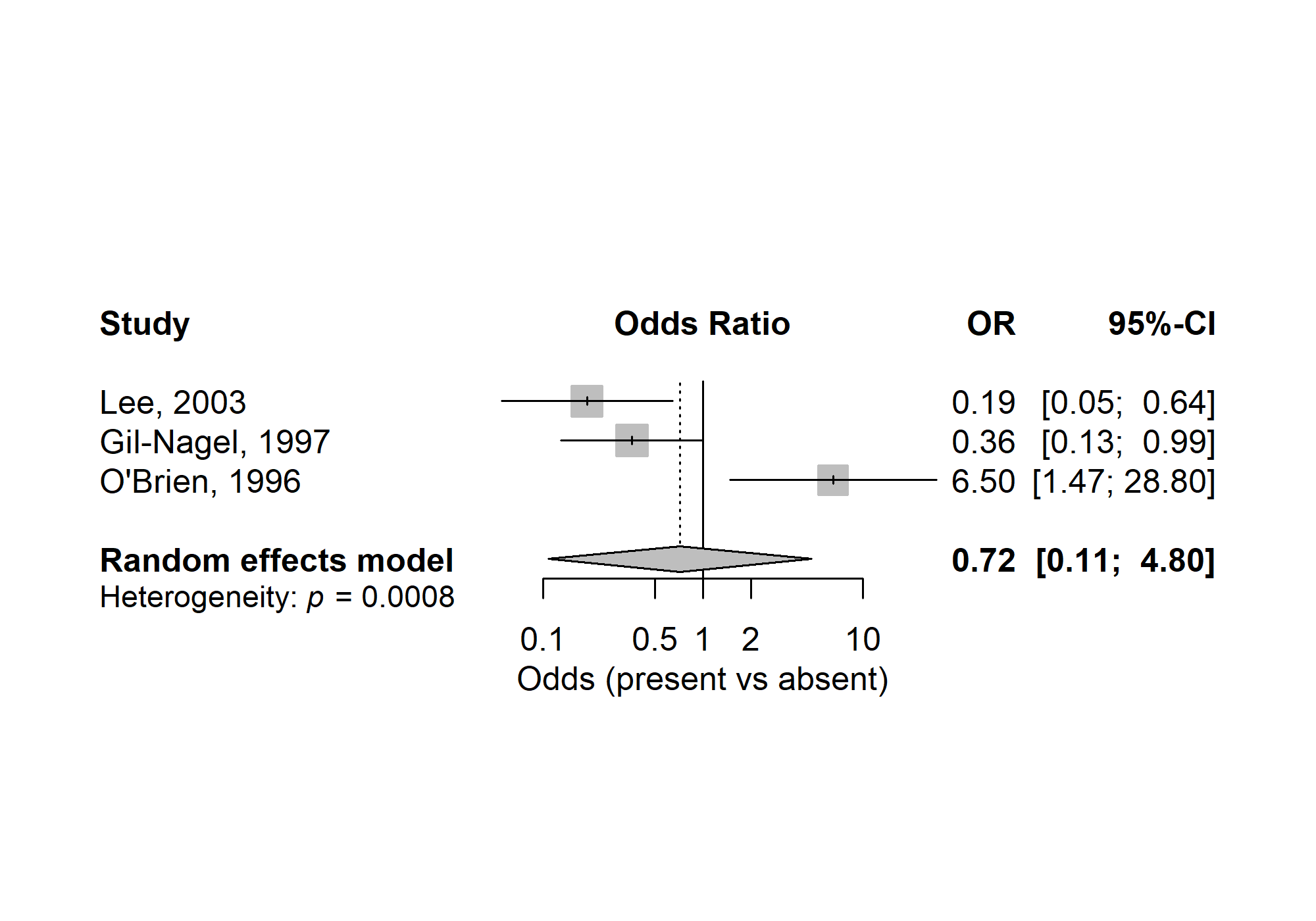


*Figure S1p: Meta-analysis on speech disturbance*


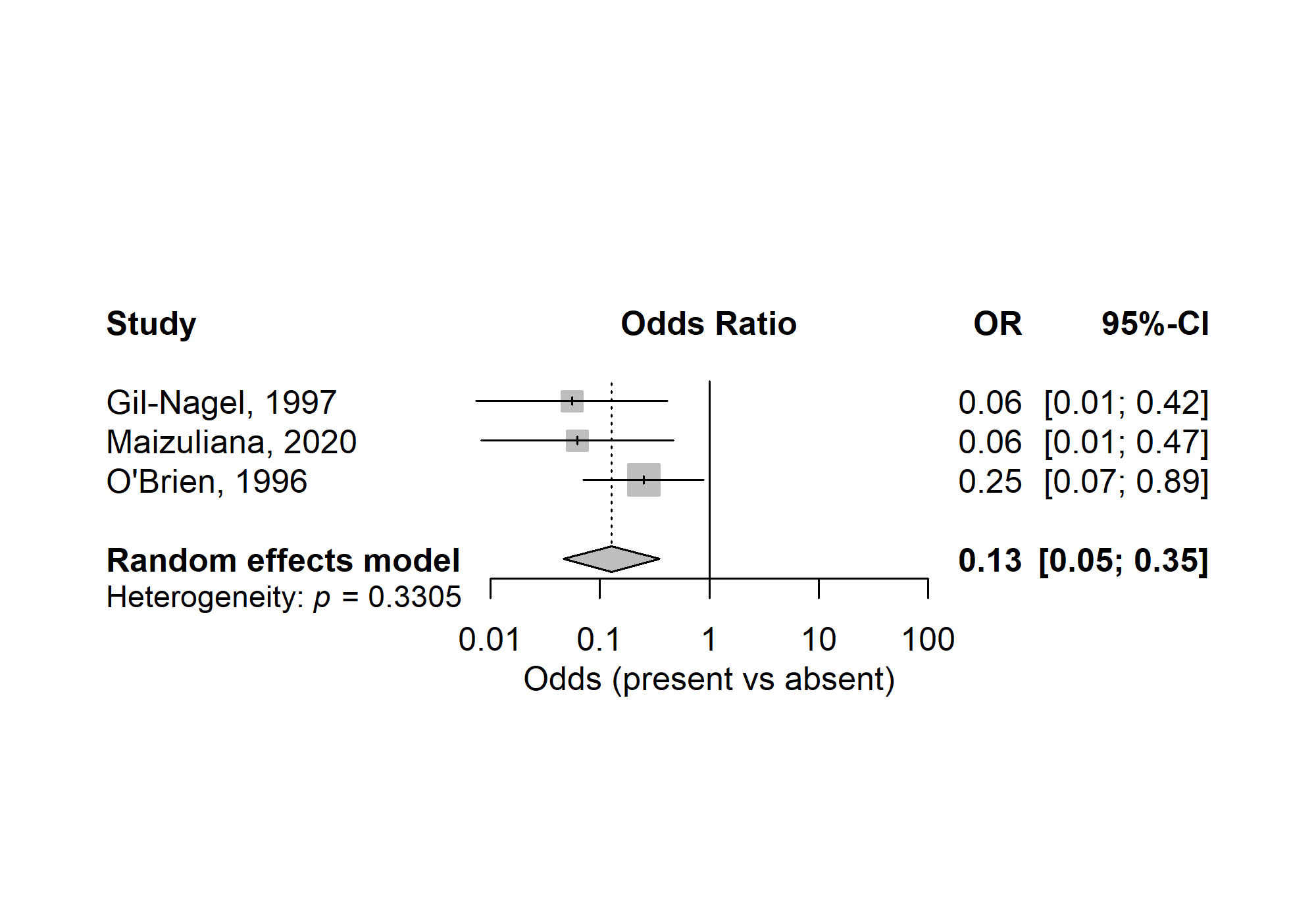


*Figure S1q: Meta-analysis on post-ictal aphasia*


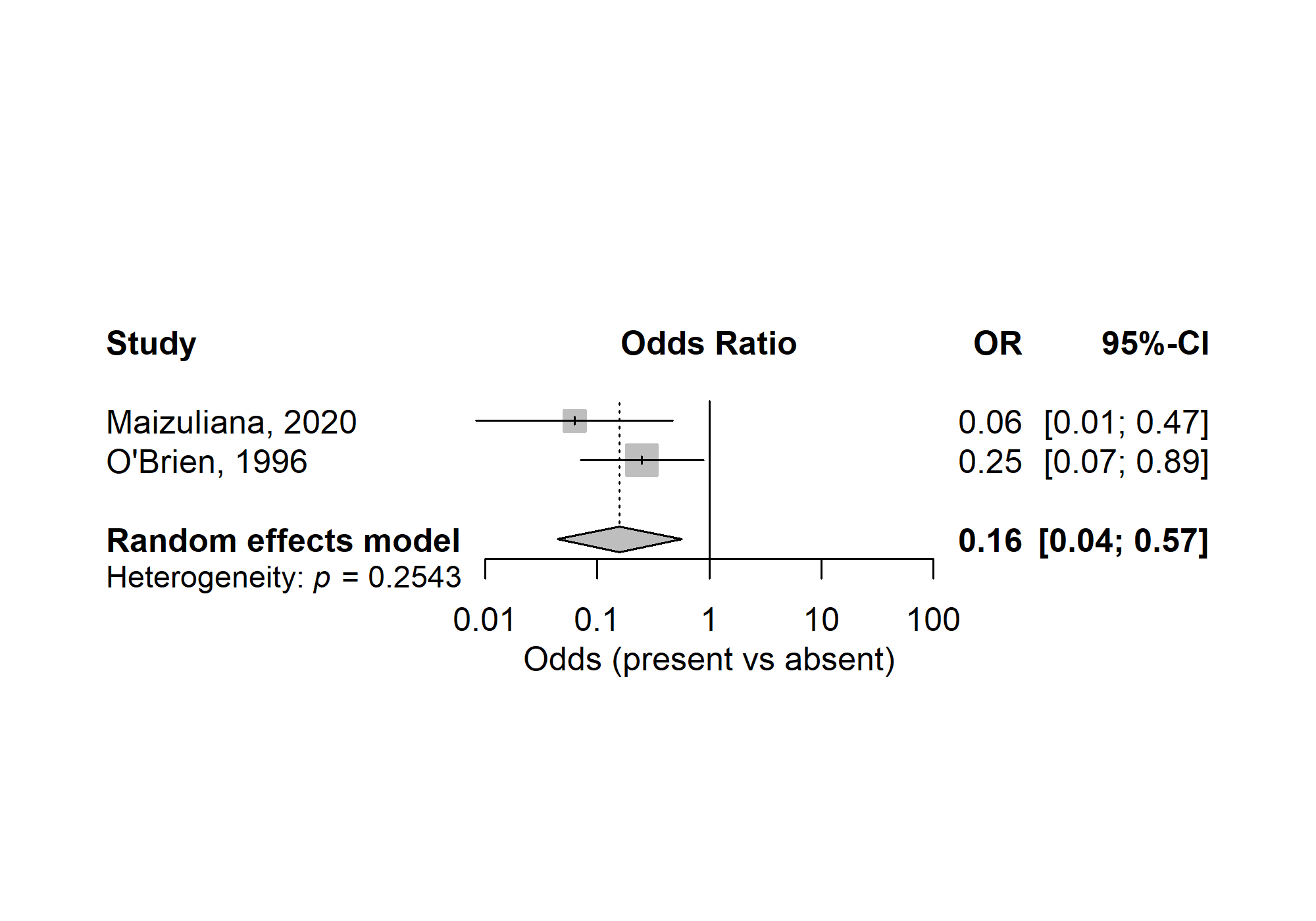


**Table S1 - Meta-analysis on the odds of occurrence of an ictal sign or symptom in lateral TLE based on a sensitivity analysis restricted to studies with >50% of patients with unequivocal lateral TLE**

| **Ictal sign or symptom** | **Assessed in  #studies (S) #patients (P)** | **Odds overall** | **Heterogeneity** |
| --- | --- | --- | --- |
| epigastric aura | 3 S / 49 P | 0.12 [95% CI 0.05-0.33] | p = 0.5016 |
| olfactory/gustatory aura | 2 S / 27 P | 0.04 [95% CI 0.01-0.29] | p = 0.6842 |
| dizziness/cephalic aura | 2 S / 30 P | 0.44 [95% CI 0.10-1.92] | p = 0.0938 |
| fear aura/psychic aura | 3 S / 49 P | 0.04 [95% CI 0.01-0.18] | p = 0.9109 |
| manual automatisms | 2 S / 41 P | 1.02 [95% CI 0.35-3.00] | p = 0.0910 |
| arrest | 2 S / 41 P | 0.25 [95% CI 0.12-0.56] | p = 0.3141 |

S, studies; P, patients; GTCS, generalized tonic-clonic seizure CI, confidence interval

Odds overall = summary estimate of the odds, resulting from the meta-analysis. Values > 1 indicate that the occurrence of an ictal sign or symptom is more likely than the absence of an ictal sign or symptom in lateral TLE. Values < 1 indicate that the absence of an ictal sign or symptom is more likely than the occurrence of an ictal sign or symptom in lateral TLE

Test for heterogeneity: if p < 0.05 -> study-specific estimates of the odds are heterogeneous, so the overall summary estimate has to be interpreted with some caution

**Figure S2 - Meta-analysis on the odds of occurrence of an ictal sign or symptom in lateral TLE based on a sensitivity analysis restricted to studies with >50% of patients with unequivocal lateral TLE**

**
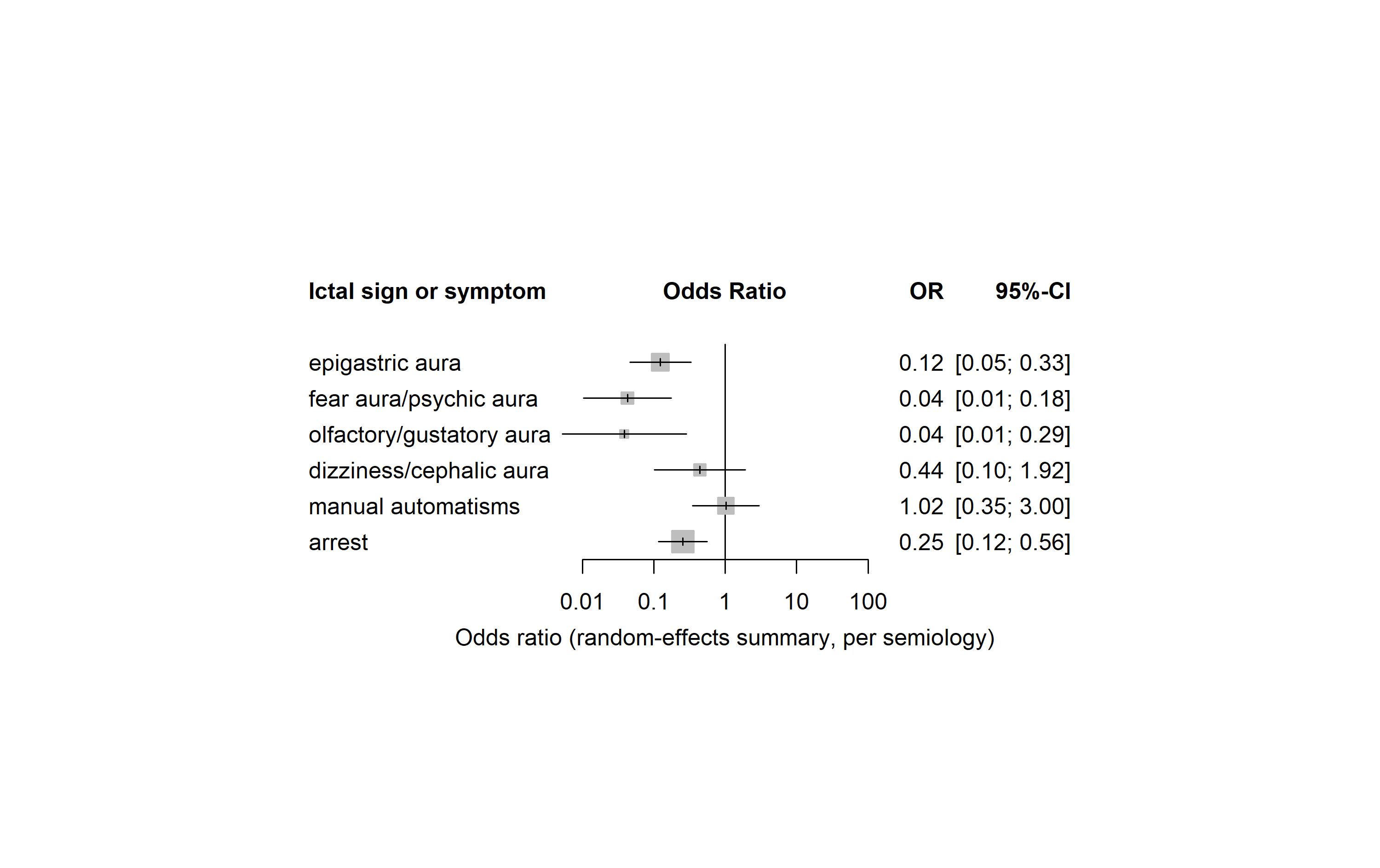
**

OR, Odds Ratio; CI, confidence interval

This forest plot shows random-effects summary ORs for the occurrence of specific ictal signs or symptoms in patients with lateral TLE. An OR > 1 indicates that the sign or symptom is more likely to be present than absent, whereas an OR < 1 indicates that the sign or symptom is more likely to be absent than present.

*Figure S2a: Meta-analysis on epigastric aura*

*
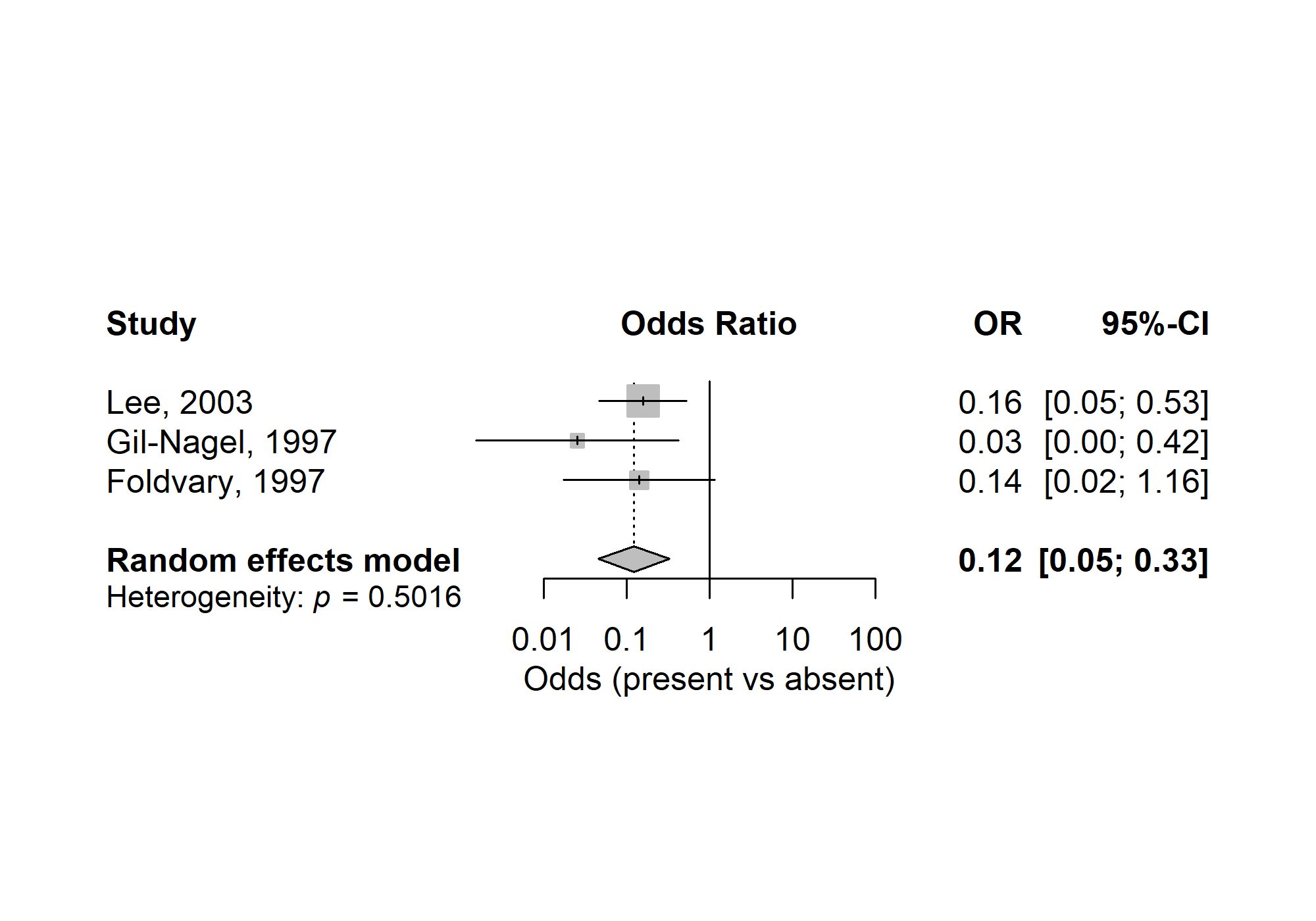
*

*Figure S2b: Meta-analysis on olfactory/gustatory aura*

*
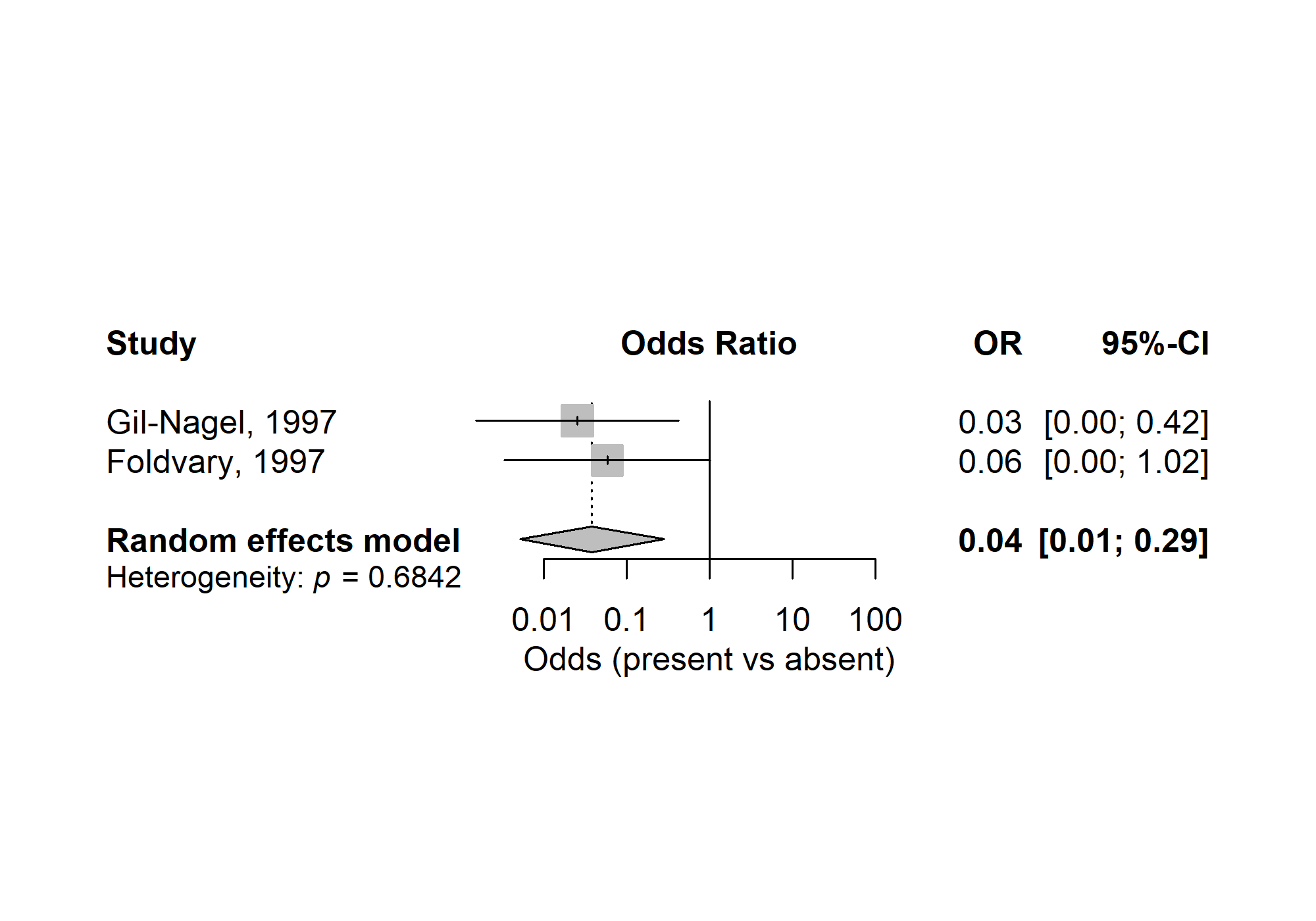
*

*Figure S2c: Meta-analysis on dizziness/cephalic aura*

*
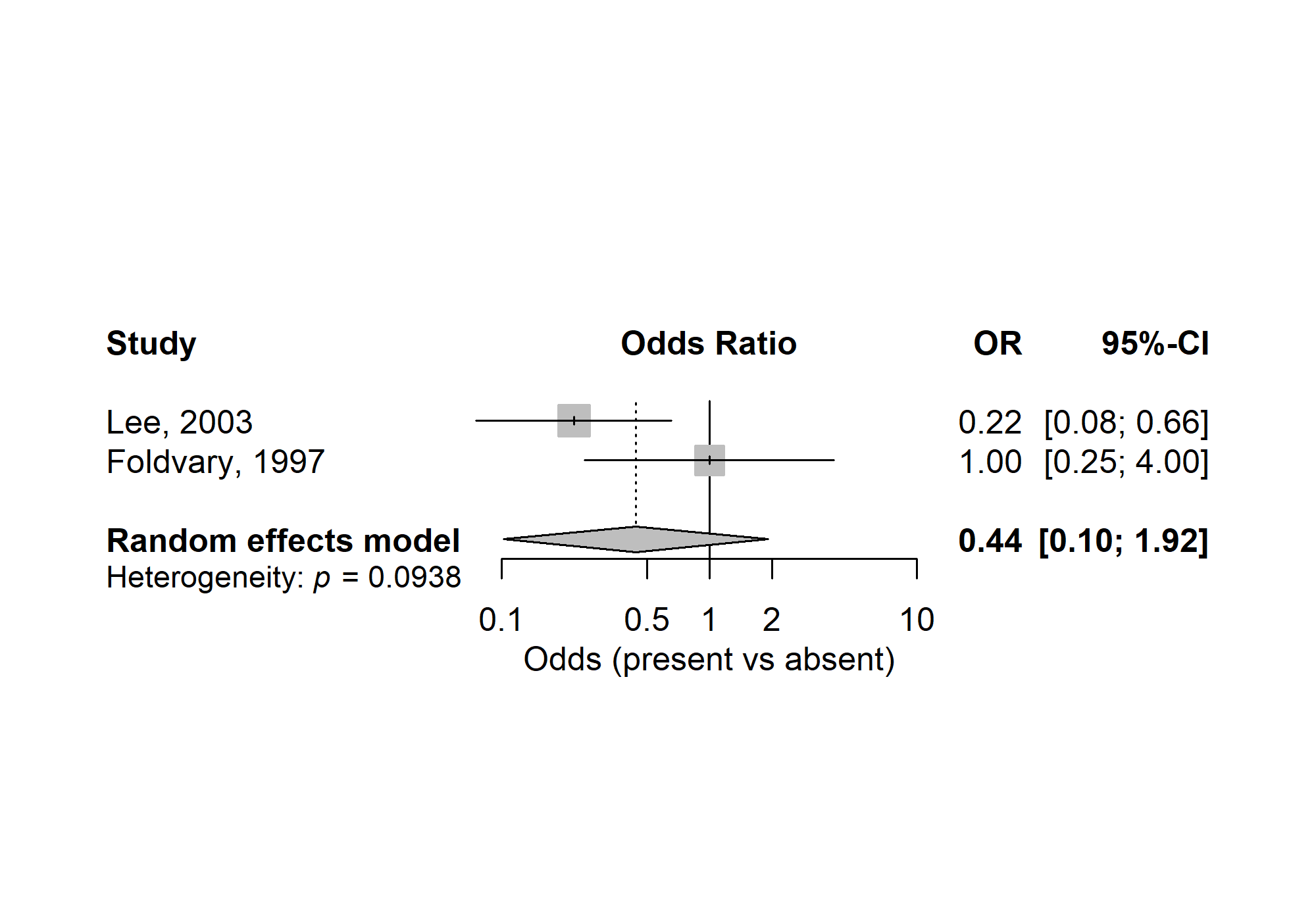
*

*Figure S2d: Meta-analysis on fear aura/psychic aura*

*
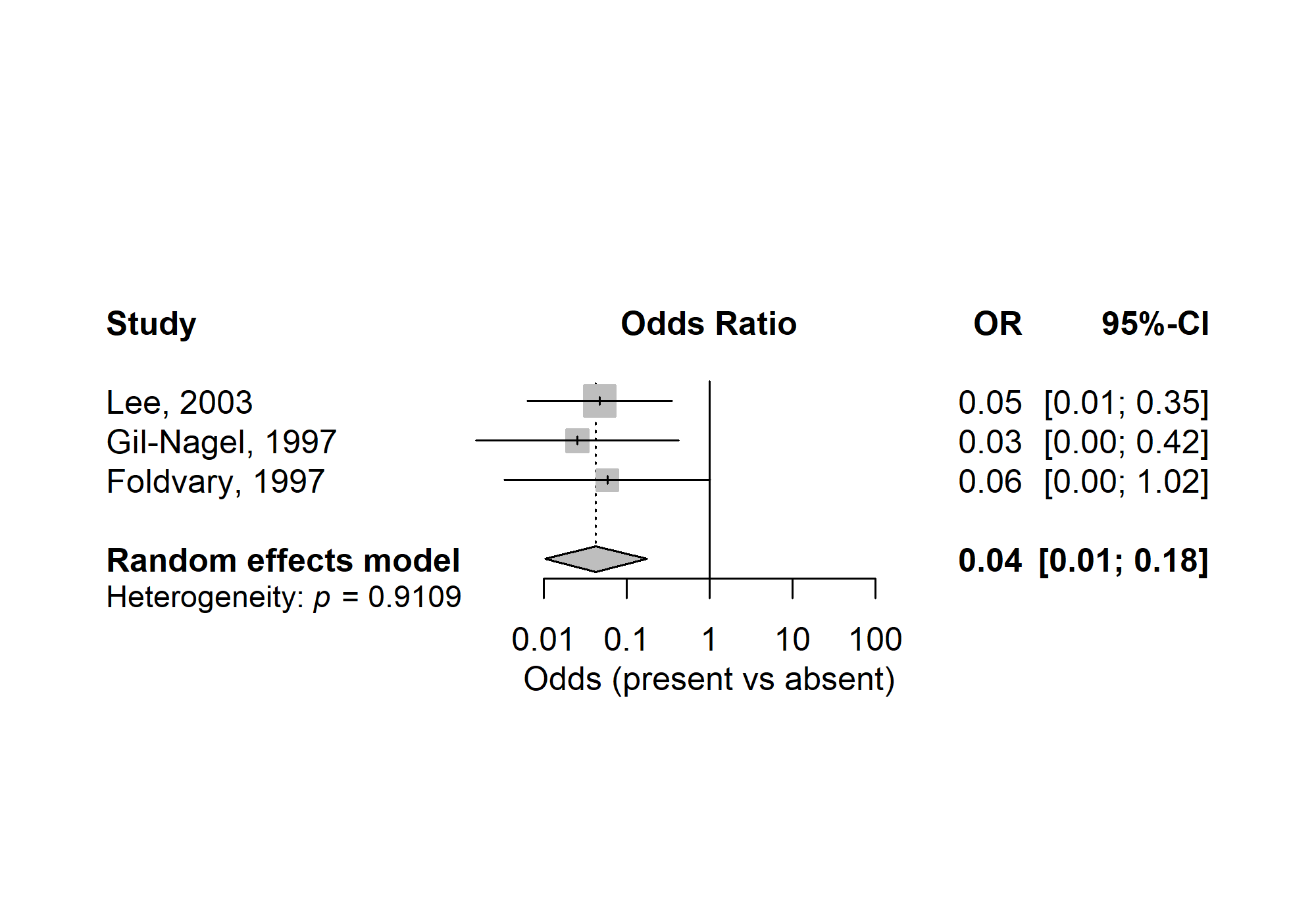
*

*Figure S2e: Meta-analysis on manual automatisms*

*
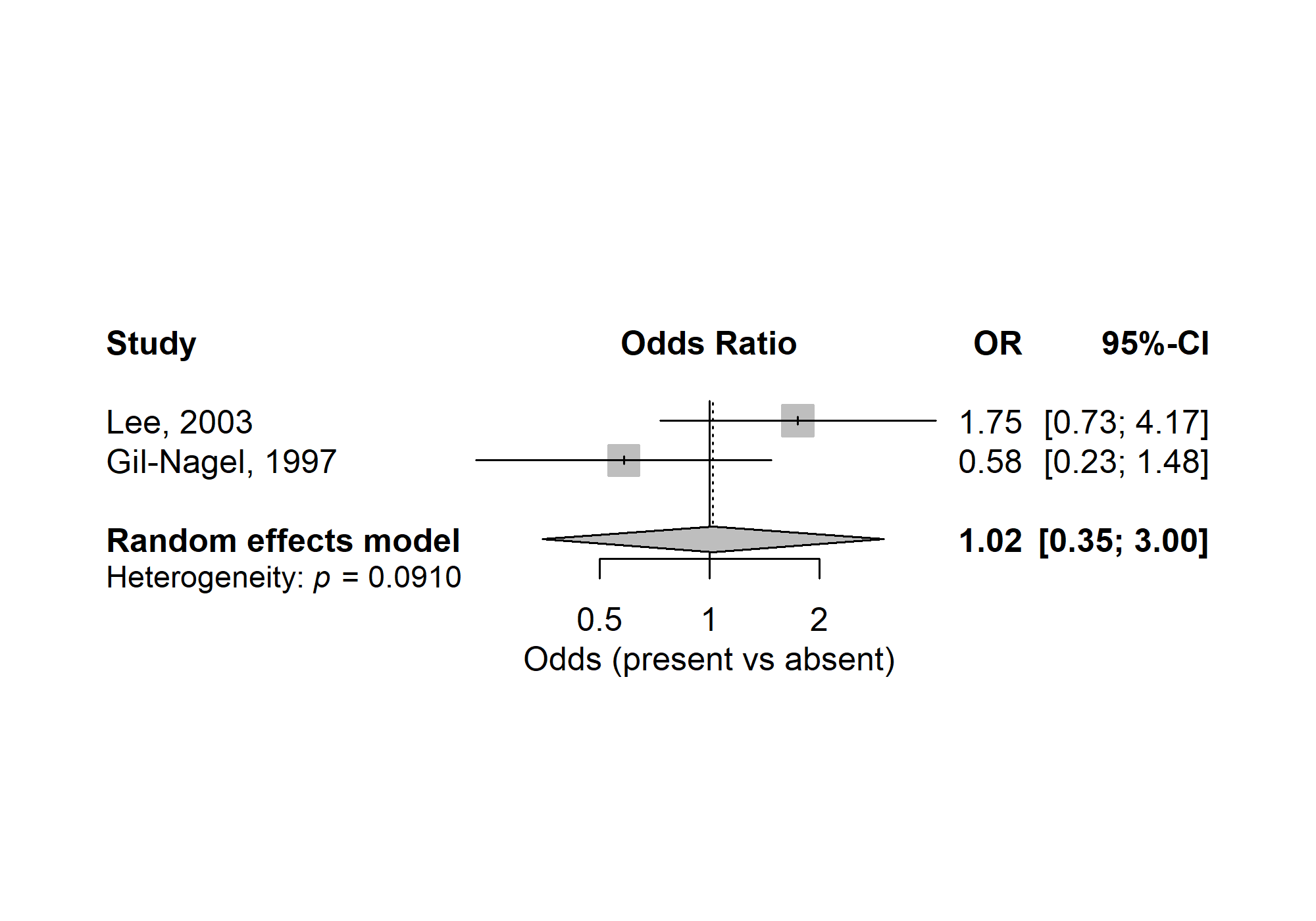
*

*Figure S2f: Meta-analysis on arrest*


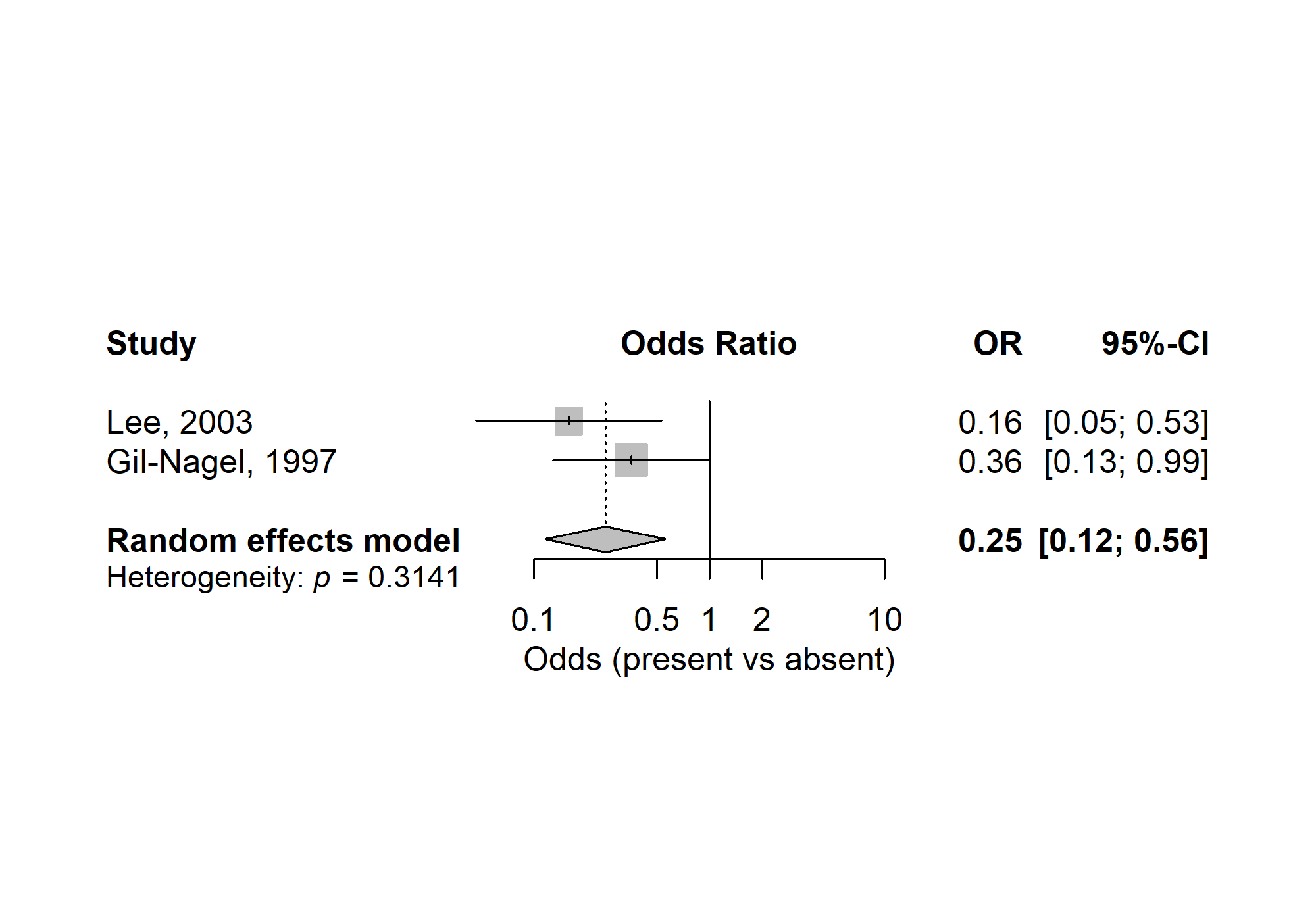


**Figure S3 - Meta-analysis on the diagnostic accuracy for differentiating lateral from mesial TLE with regards to the presence or absence of relevant semiological features**

*Figure S3a : Meta-analysis on the diagnostic accuracy of oral automatisms*

*
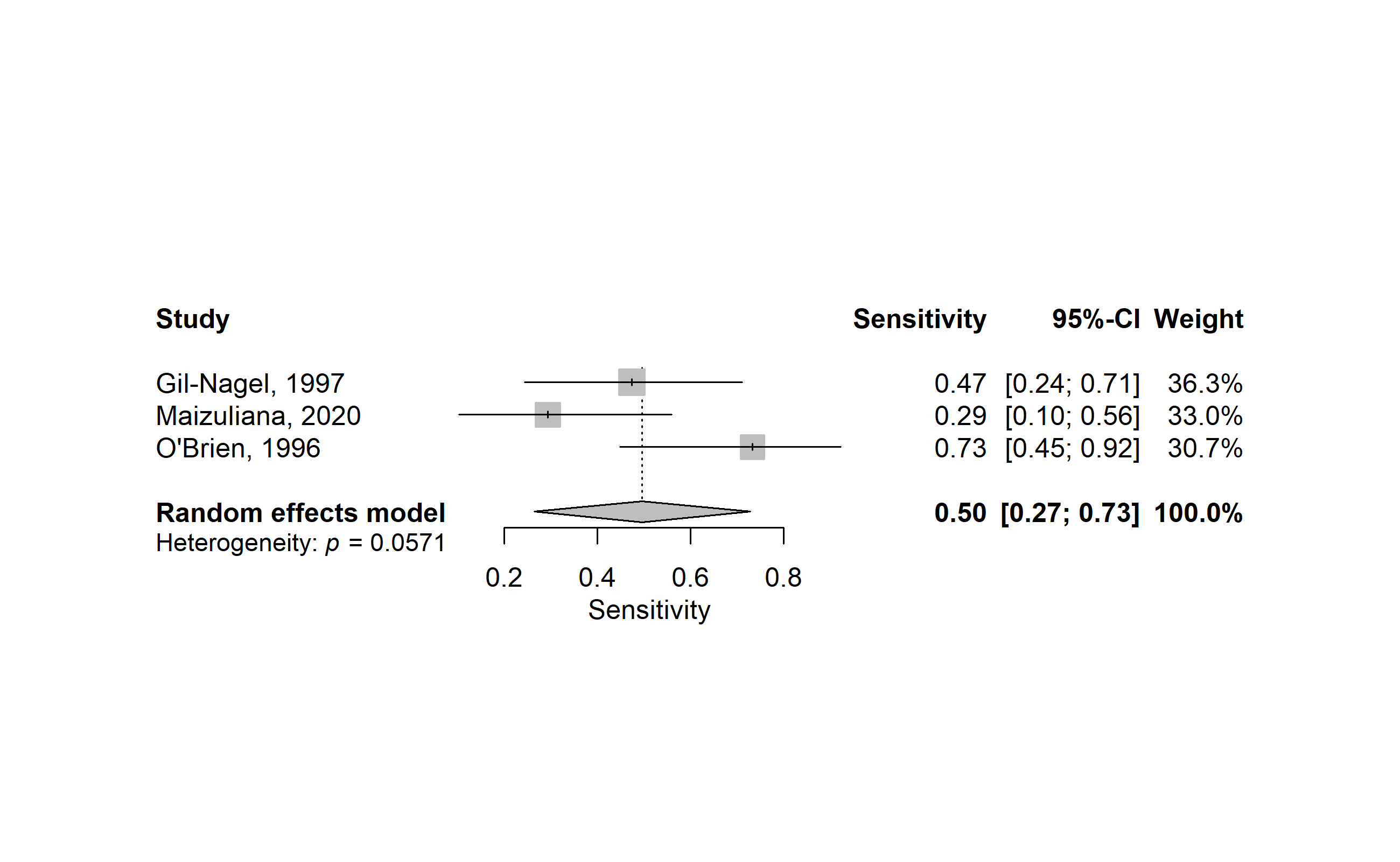

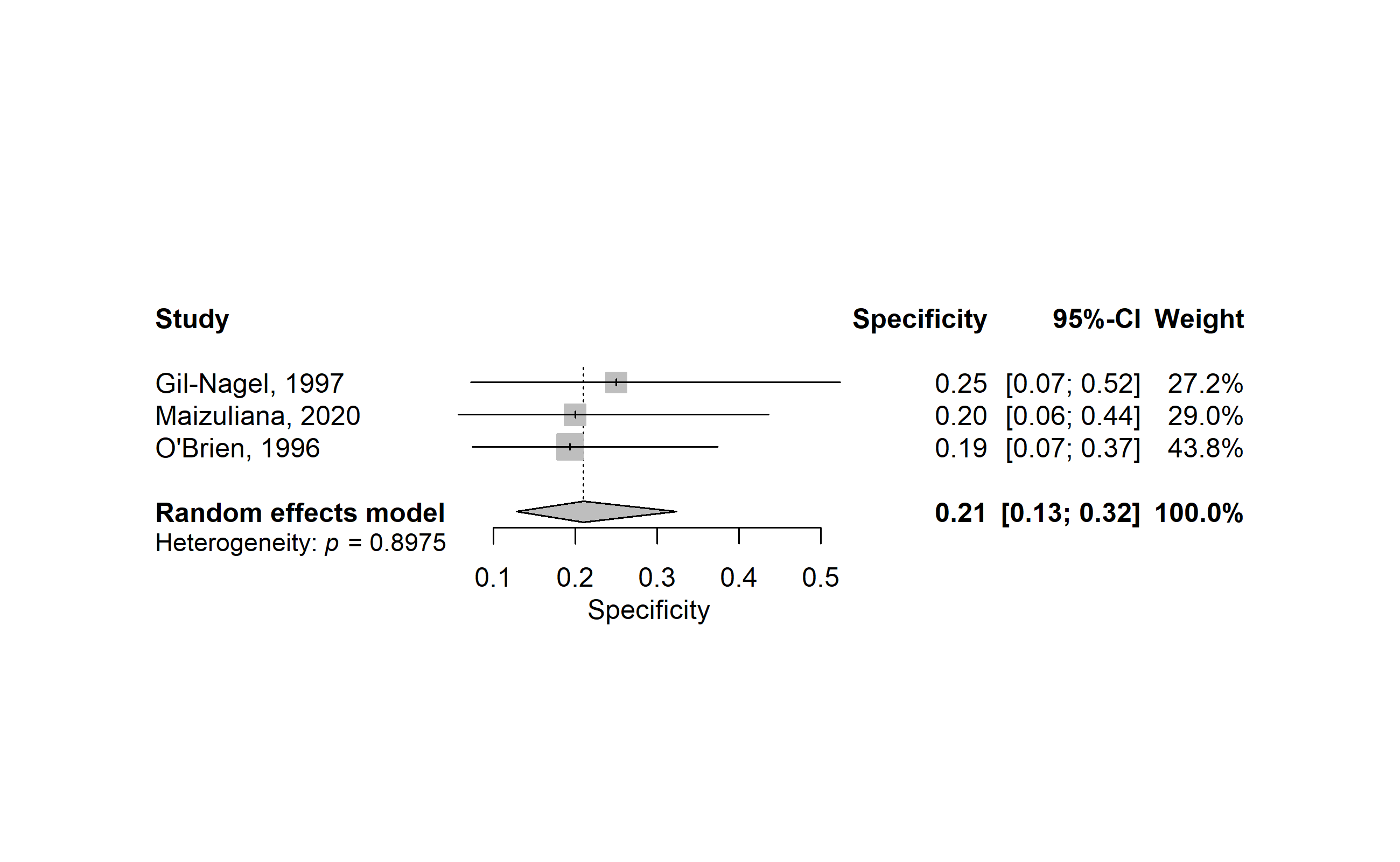
*

*Figure S3b: Meta-analysis on the diagnostic accuracy of auditory aura*

*
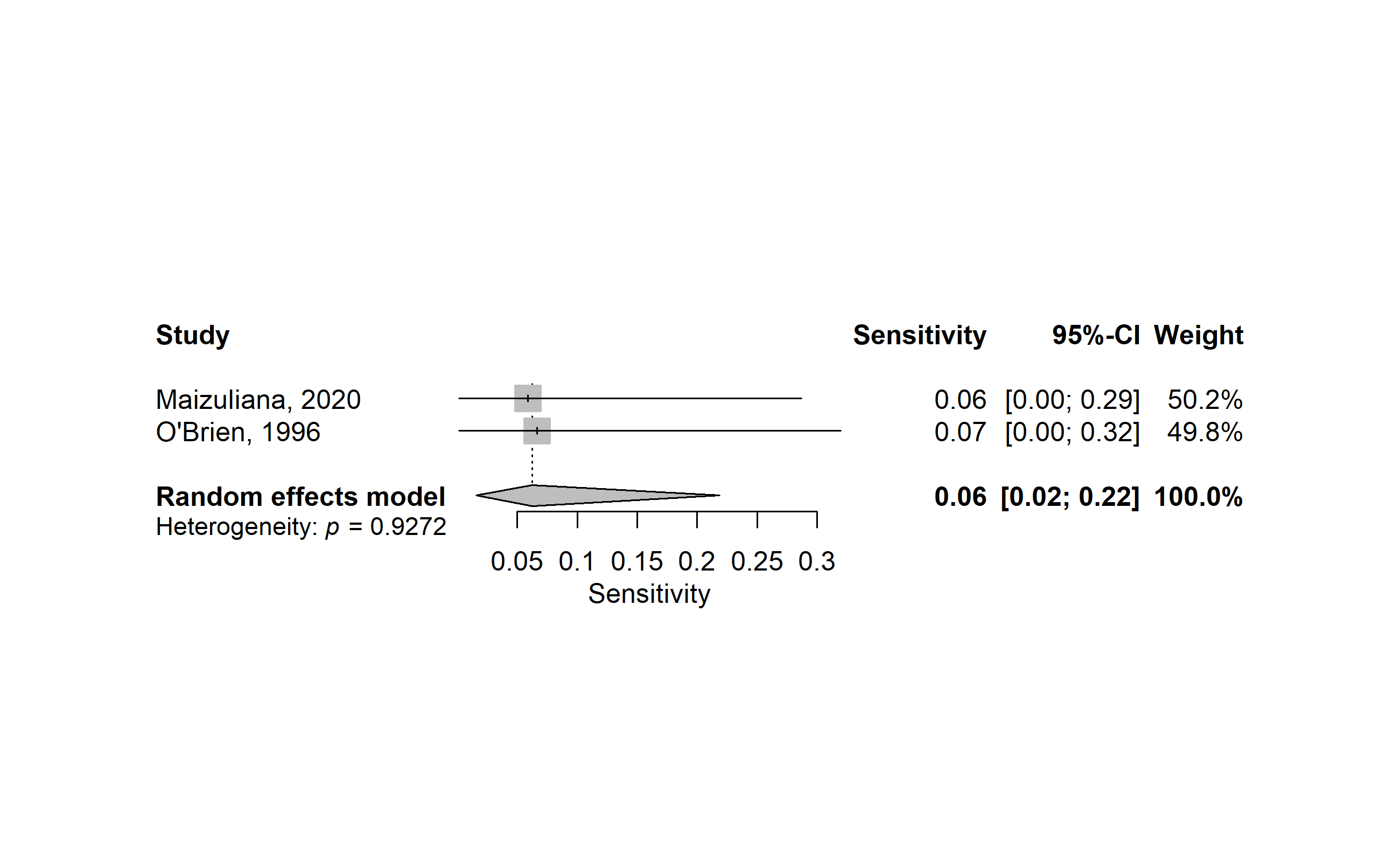

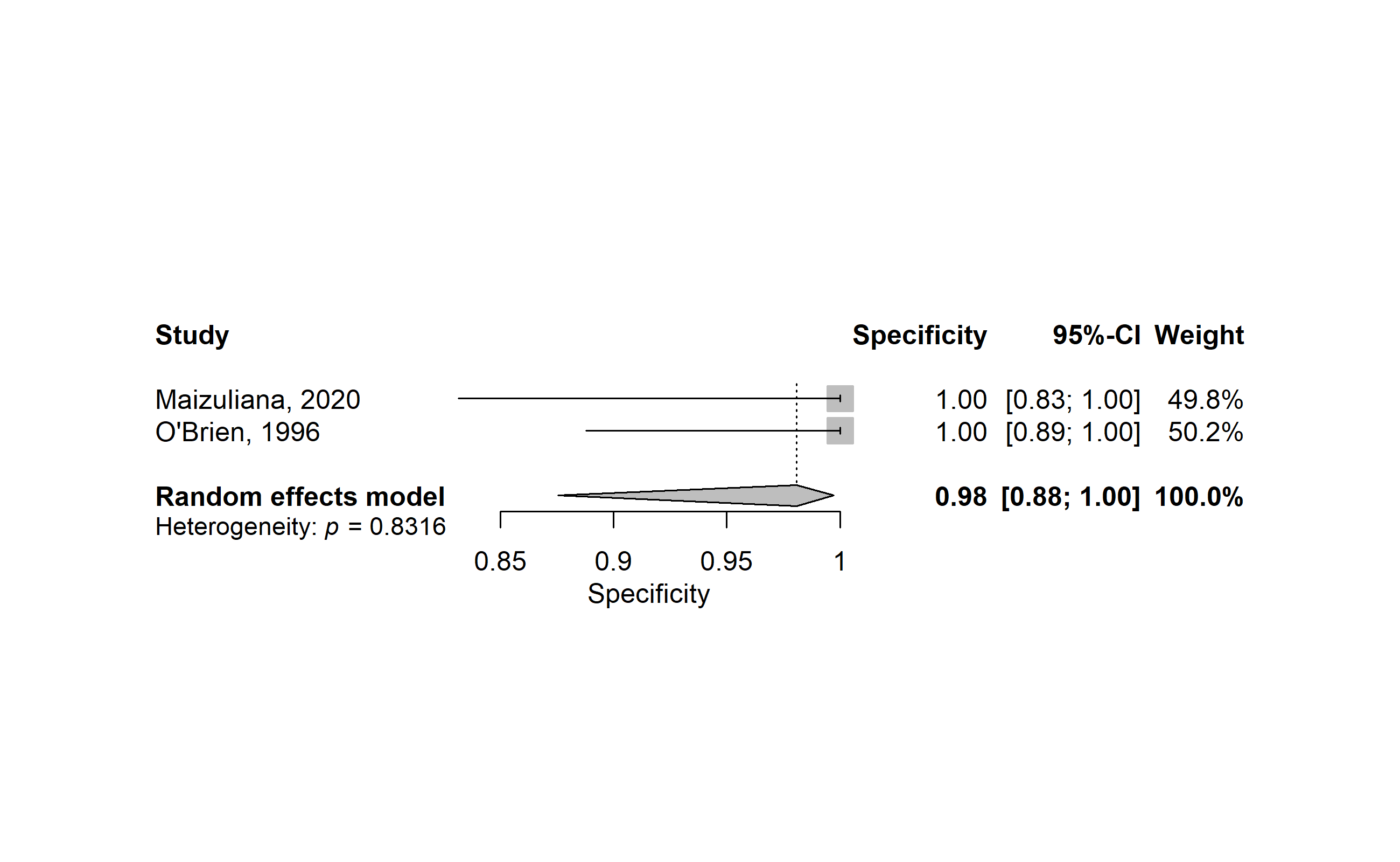
*

*Figure S3c: Meta-analysis on the diagnostic accuracy of lack of contralateral dystonic posturing*


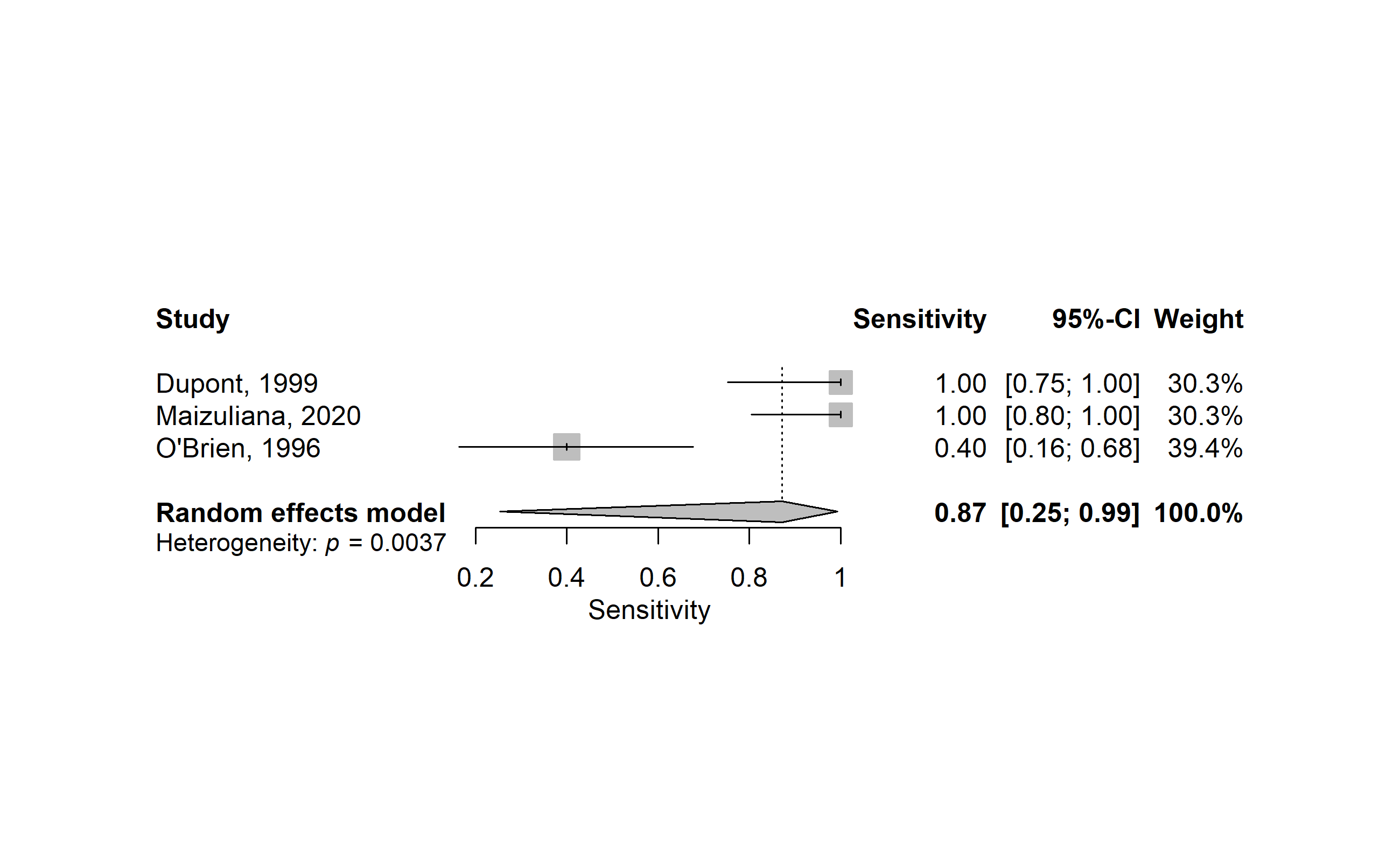


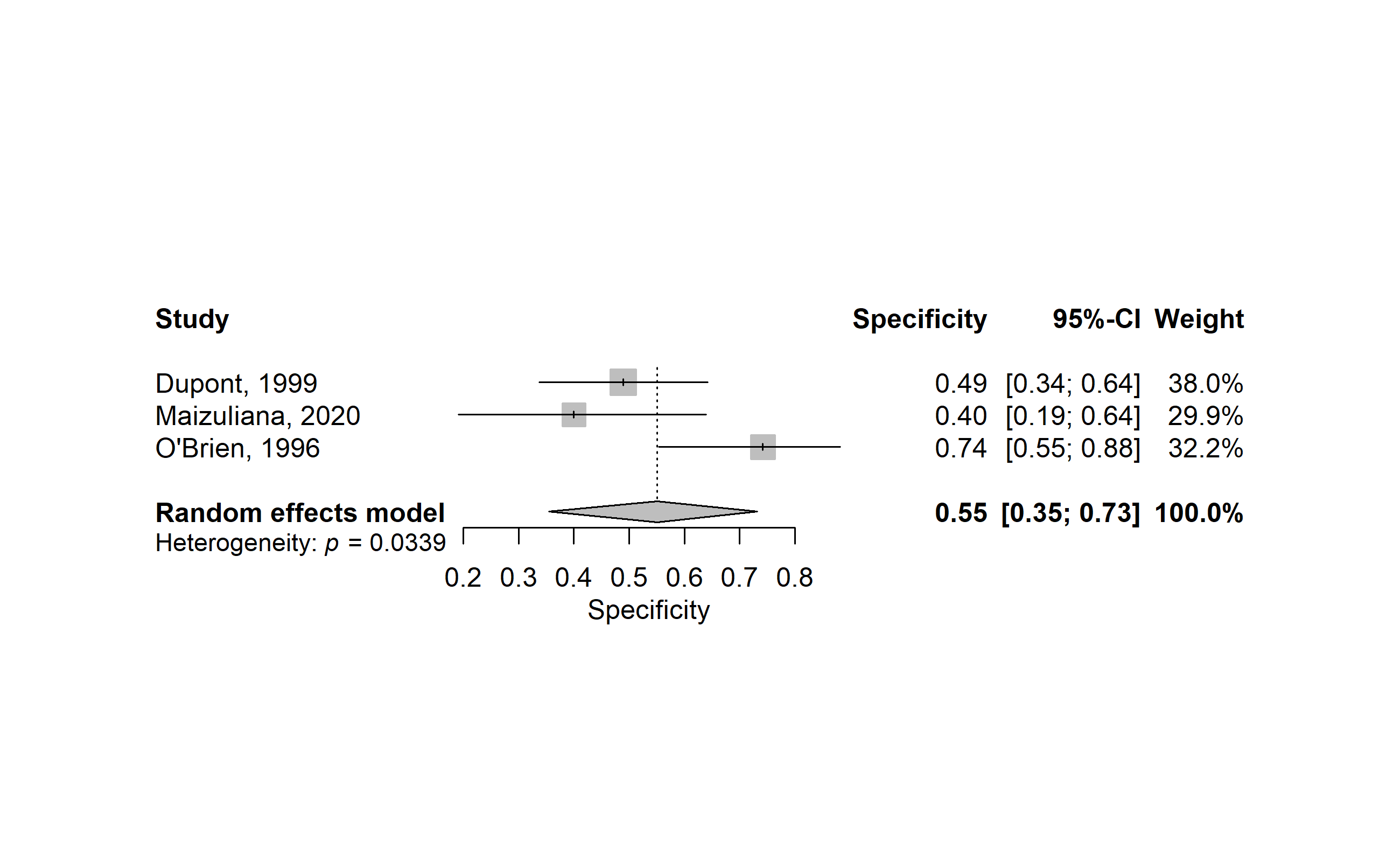


*Figure S3d: Meta-analysis on the diagnostic accuracy of lack of olfactory/gustatory aura*


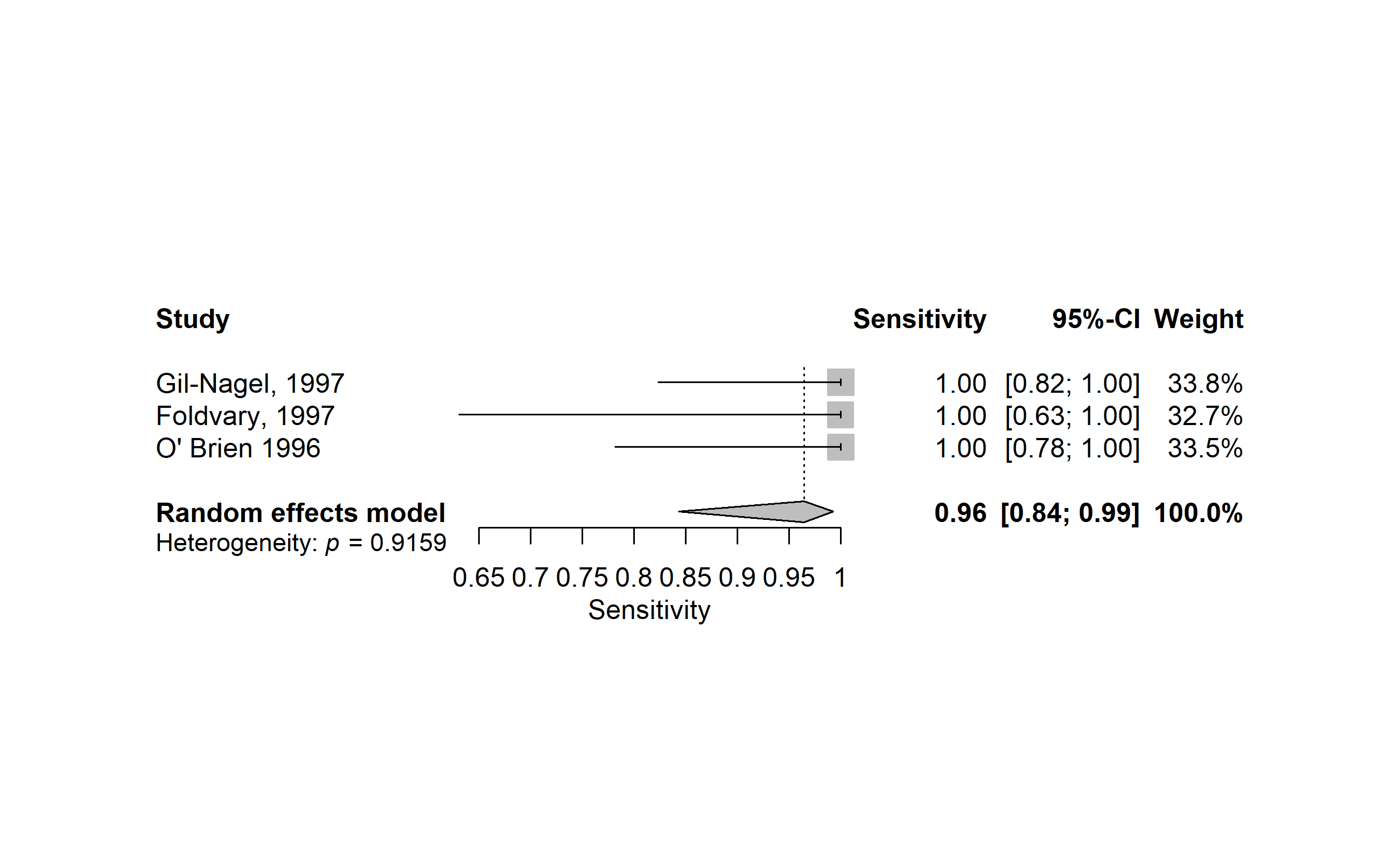

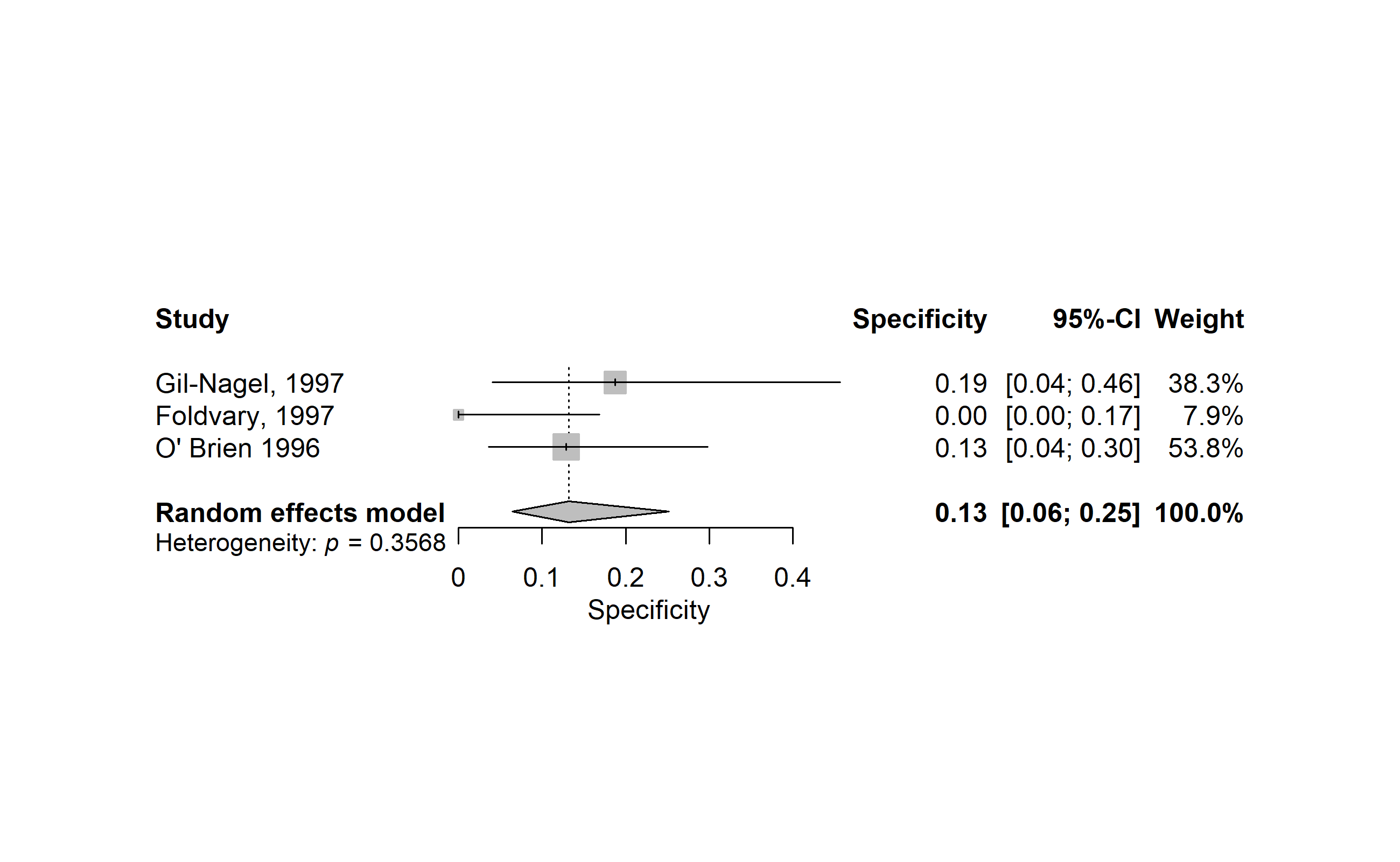


*Figure S3e: Meta-analysis on the diagnostic accuracy of manual automatisms*


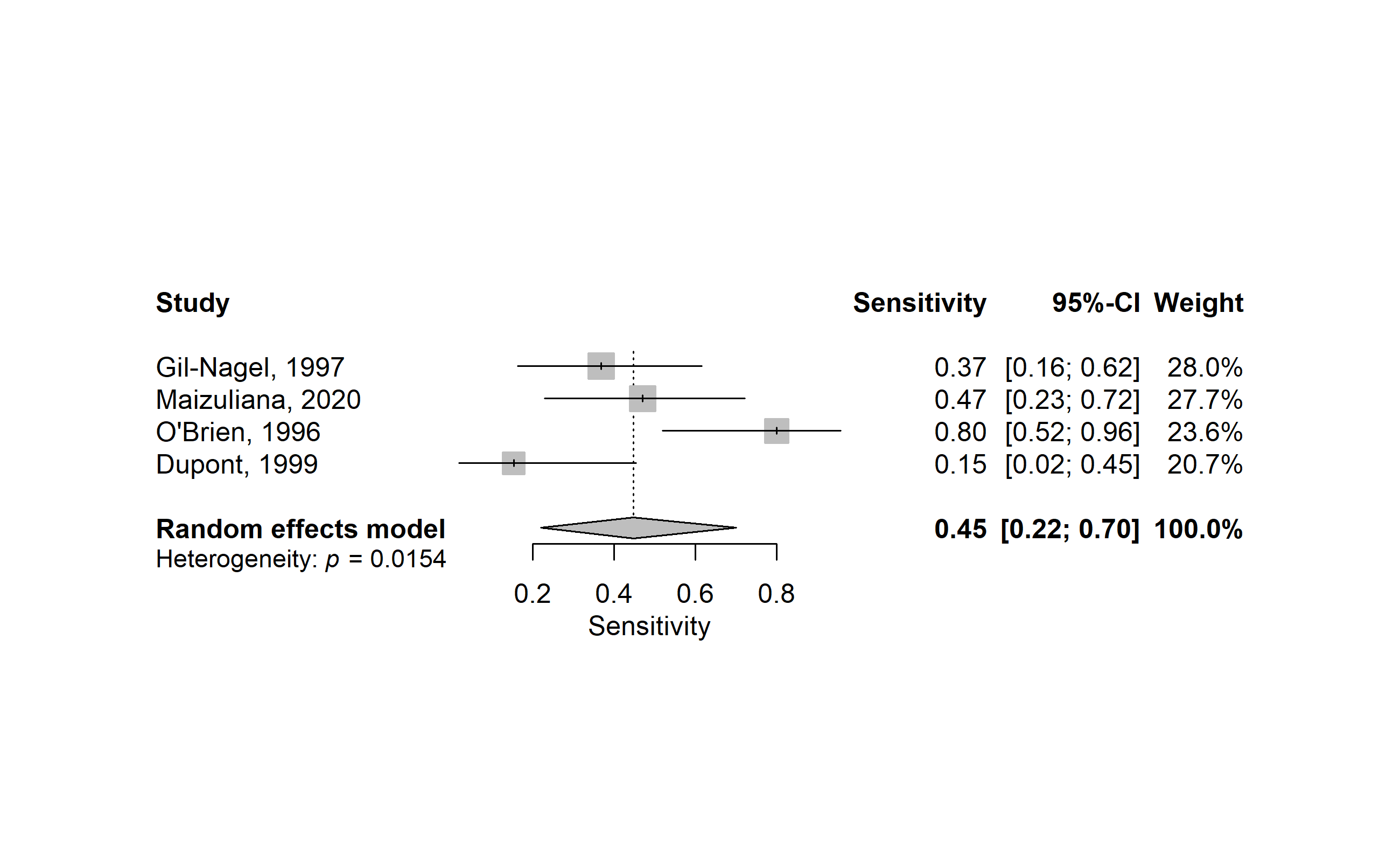

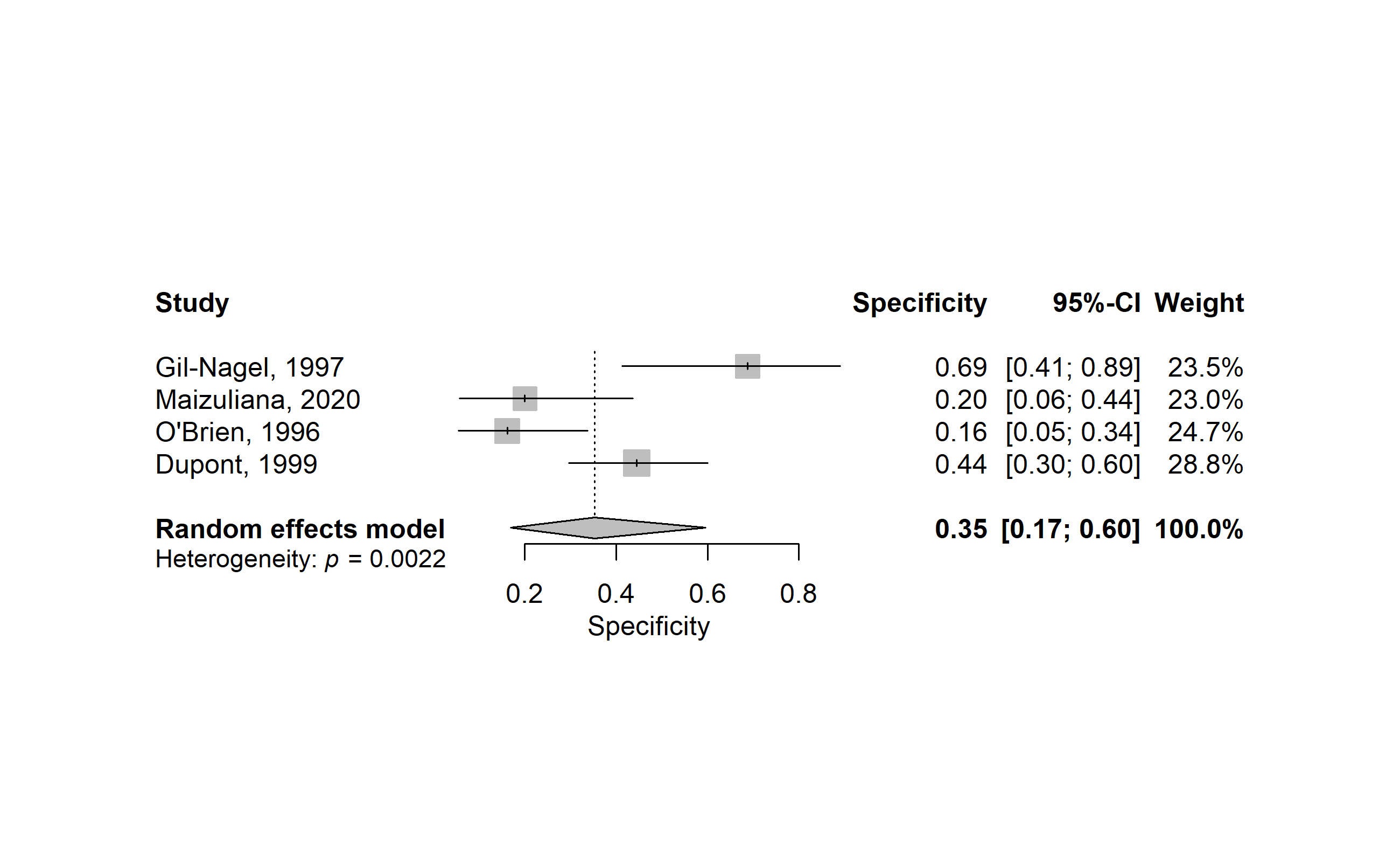


*Figure S3f: Meta-analysis on the diagnostic accuracy of arrest reactions*


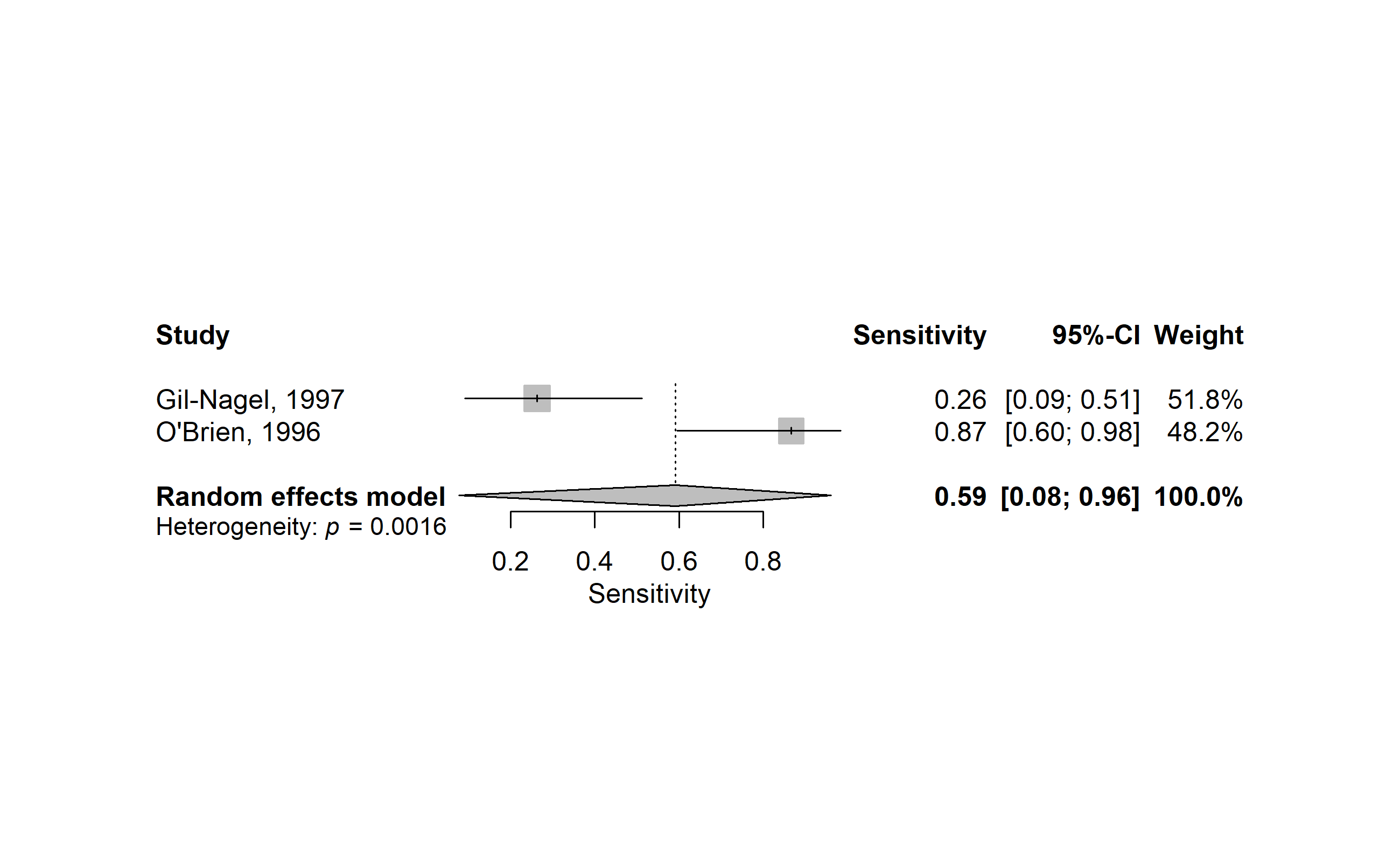

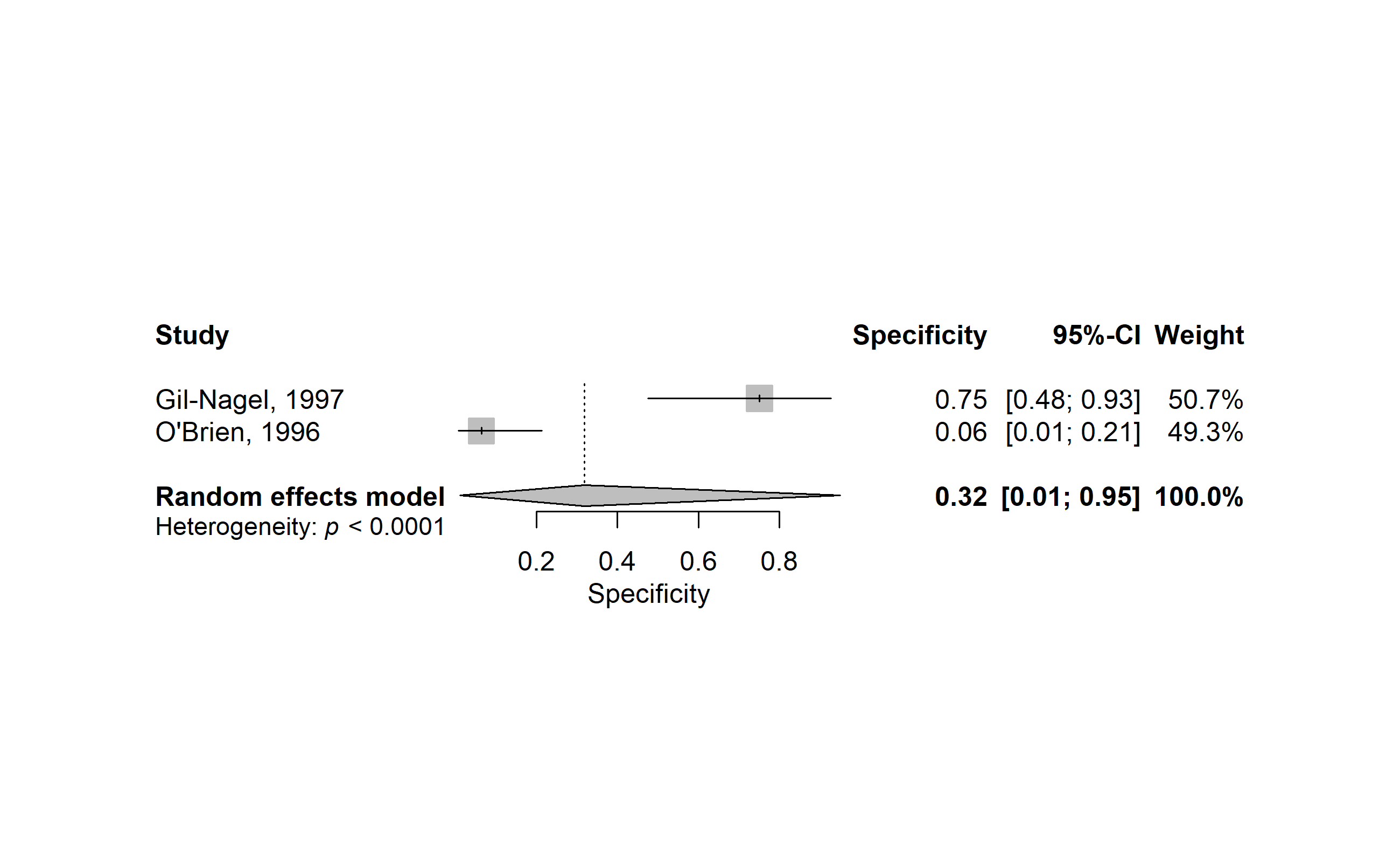


*Figure S3g: Meta-analysis on the diagnostic accuracy of lack of epigastric aura
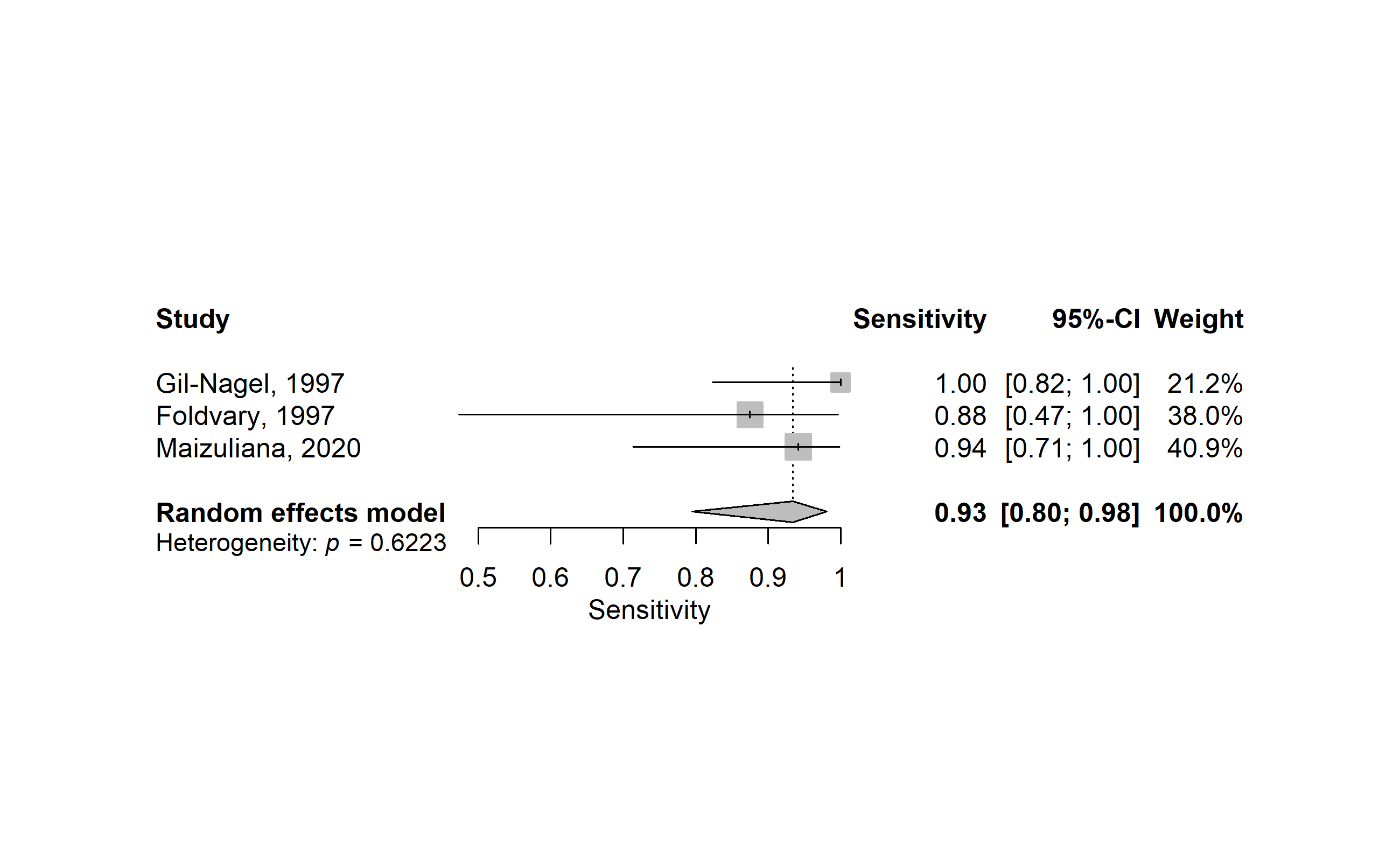

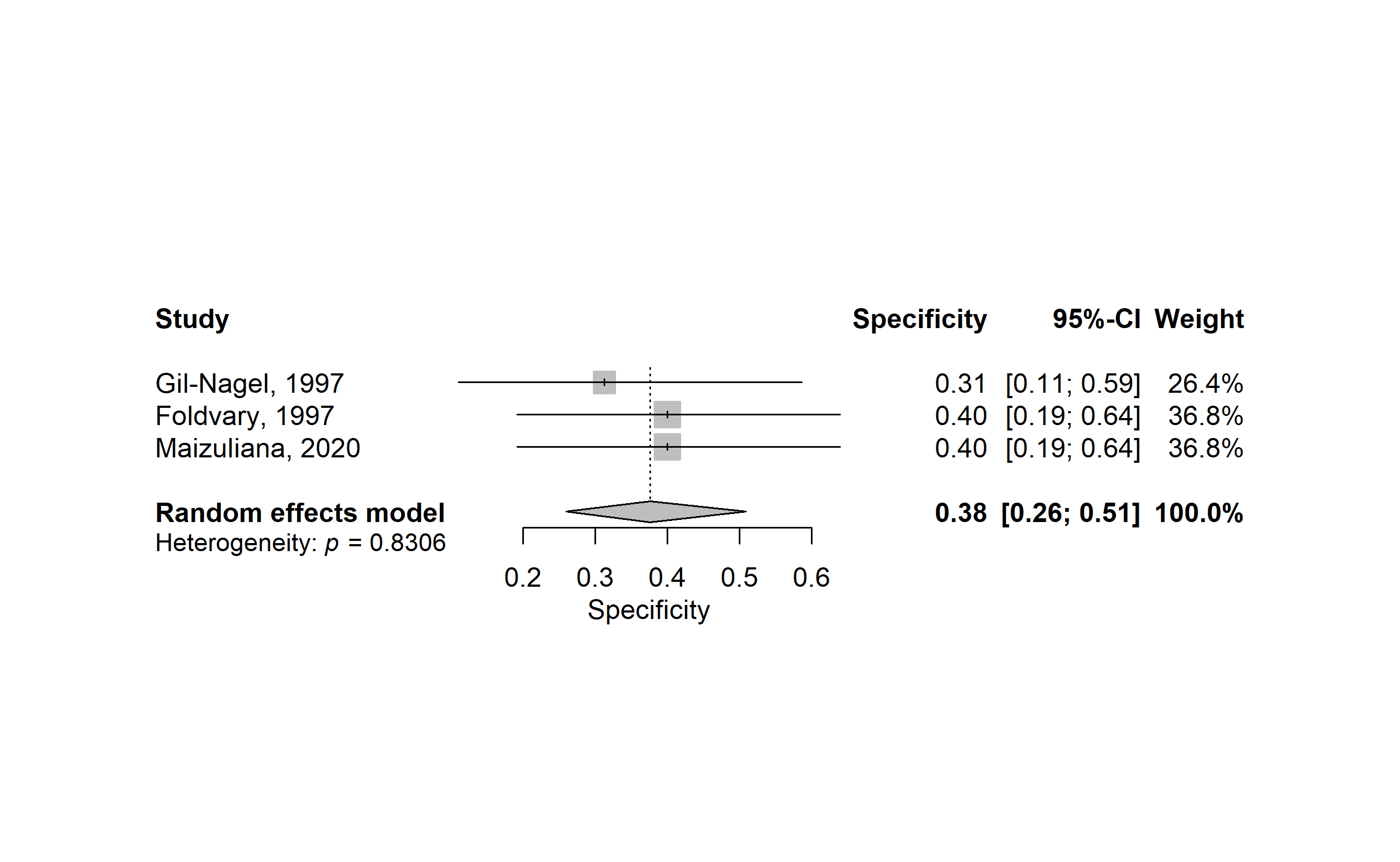
*
