# Supplementary material for: Ictal semiology in lateral temporal epilepsy: A systematic review and meta‐analysis
Source: Epileptic Disord. 2026 Feb 2;28(3):678–89. doi: 10.1002/epd2.70189 (PMC13276704; doi:10.1002/epd2.70189)
Supplement: Supplementary file 3 — Appendix S1. [file EPD2-28-678-s002.docx]

**TEST YOURSELF**

**Answers**

**1**. **D**

**2. D**

**3. B**
